# Tropical cyclones: what are their impacts on phytoplankton ecology?

**DOI:** 10.1093/plankt/fbac062

**Published:** 2022-11-21

**Authors:** Peter A Thompson, Hans W Paerl, Lisa Campbell, Kedong Yin, Karlie S McDonald

**Affiliations:** CSIRO Oceans and Atmosphere, 4-5 Castray Esplanade, Hobart, 7000, Tasmania, Australia; Institute of Marine Sciences, University of North Carolina at Chapel Hill, 3431 Arendell Street, Morehead City, NC 28557, USA; Department of Oceanography, MS-3146, Texas A&M University, College Station, TX 77843, USA; Southern Marine Science and Engineering Guangdong Laboratory (Zhuhai), and School of Marine Sciences, Sun Yat-Sen University, University Road 2, Zhuhai, 519082, China; Fisheries and Aquaculture Centre, Institute for Marine and Antarctic Studies, University of Tasmania, 15-21 Nubeena Crescent, Taroona, 7053, Tasmania, Australia

**Keywords:** cyclone, hurricane, phytoplankton, ecology

## Abstract

Following the passage of a tropical cyclone (TC) the changes in temperature, salinity, nutrient concentration, water clarity, pigments and phytoplankton taxa were assessed at 42 stations from eight sites ranging from the open ocean, through the coastal zone and into estuaries. The impacts of the TC were estimated relative to the long-term average (LTA) conditions as well as before and after the TC. Over all sites the most consistent environmental impacts associated with TCs were an average 41% increase in turbidity, a 13% decline in salinity and a 2% decline in temperature relative to the LTA. In the open ocean, the nutrient concentrations, cyanobacteria and picoeukaryote abundances increased at depths between 100 and 150 m for up to 3 months following a TC. While at the riverine end of coastal estuaries, the predominate short-term response was a strong decline in salinity and phytoplankton suggesting these impacts were initially dominated by advection. The more intermediate coastal water-bodies generally experienced declines in salinity, significant reductions in water clarity, plus significant increases in nutrient concentrations and phytoplankton abundance. These intermediate waters typically developed dinoflagellate, diatom or cryptophyte blooms that elevated phytoplankton biomass for 1–3 months following a TC.

## INTRODUCTION

Severe weather associated with tropical cyclones (TCs) can cause devastating loss of life, significant infrastructure damage and intense or widespread flooding, but their impacts on many aspects of aquatic ecology remain poorly quantified. In the open ocean, the basic impacts of TCs on phytoplankton ecology are increasingly detected and characterized by remote sensing (e.g. Babin *et al.,* 2004; Zhao *et al.,* 2008; Chen *et al.,* 2017; Pan *et al.,* 2018) with a rise in green reflectance indicative of increased near surface chlorophyll *a* (chl*a*). However, in the open ocean the rapid rise in surface chl*a* may simply reflect the vertical mixing of a deep chl*a* maximum closer to the surface while the effects of TCs on planktonic biodiversity, primary production (PP) and trophic transfer remain largely unknown. In the coastal zone (rivers, estuaries and shelf regions) conditions associated with TCs can cause extremely high winds, tidal surge, large waves, increased vertical and horizontal mixing, significant rainfall, runoff, nutrient inputs and reduced irradiance. To date the impacts of TCs on phytoplankton ecology have been reported from relatively few locations and events (e.g. Tsuchiya *et al.,* 2014; Anglès *et al.,* 2015; Paerl *et al*., 2018, 2020).

TCs are relatively rare in time and space, almost always arise between 5° and 30° of latitude, and are increasingly moving poleward (Murakami *et al.,* 2013; Altman *et al.,* 2018). In the northern hemisphere, the average number of TCs is 38 y^−1^ (Knaff *et al.,* 2014), and the most frequently impacted locations are: China, Philippines, Japan, Mexico, United States of America, Taiwan, Vietnam, India and Cuba. The southern hemisphere experiences an average of 13 y^−1^ primarily impacting Australia and eastern Africa including Madagascar (Pillay and Fitchett, 2020). There is a strong seasonality in TC occurrence from being almost absent from mid-winter to mid-spring and reach their peak frequency in late summer and early autumn (Knaff *et al.,* 2014). Considerable debate exists regarding whether the frequency of TCs are increasing due to a warming climate (Allan and Soden, 2008; Knutson *et al.,* 2010; Seneviratne *et al.,* 2012); however, there is general agreement that TC intensity and associated precipitation are likely to rise on the order of 7% for every 1°C increase in temperature (Westra *et al.,* 2014).

PP is one of the world’s most important biogeochemical processes and ~50% of it is by phytoplankton (Field *et al.,* 1998; Malone *et al.,* 1999; Sigman and Hain, 2012). This PP sets the upper limit on the biomass of marine ecosystems and the portion that sinks below the ocean’s surface mixed layer is important as a reservoir of carbon. The PP of the ocean frequently becomes nutrient limited especially during late summer and early autumn—peak TC season. TCs can inject nutrients into the euphotic zone increasing PP (Lin *et al.,* 2003) and rapidly cooling the surface mixed layer thereby increasing the annual CO_2_ efflux significantly (Bates *et al.,* 1998). Open ocean studies using remotely sensed data report increased surface chl*a* in the days following TC passage; essentially as soon as the skies are clear enough to reliably detect a signal. The rapid response in surface chl*a*, and its positive relationship to wind speed plus mixed layer depth (Babin *et al.,* 2004; Zhao *et al.,* 2009; Pan *et al.*, 2018), suggest that low pressure and the extreme winds associated with TCs impact phytoplankton ecology by reducing stratification, mixing a deep chl*a* maximum upwards, shallowing the nutricline or spawning an upwelling eddy. The spatial scale of the impacts is significant with analysis showing increased chl*a* within ±150 km of a TC path (Lin and Oey, 2016) resulting in an estimated 30% more PP across 5–30° of latitude in both hemispheres (Lin *et al.,* 2003). Increased vertical mixing is often associated with shifting the phytoplankton community balance from dinoflagellates to diatoms (c.f. Margalef, 1978; Taylor *et al.,* 1993; Wasmund *et al.,* 1998). Indeed, an increase in diatoms post TC has been indicated by ocean color (Son *et al.,* 2007), although this has not been confirmed by *in situ* open ocean studies.

With >70% of the world’s population residing in coastal watersheds (Vitousek *et al.,* 1997; Vörösmarty *et al.,* 2000), most estuaries are already under pressure from poor water quality (Boesch *et al.,* 2001; Paerl *et al.,* 2006), which is exacerbated by the effects of climate variability and change (Trenberth, 2011; Seneviratne *et al.,* 2012; IPCC, 2013; Paerl *et al.*, 2018, 2020). Large watersheds discharging to estuaries can act as concentrating mechanisms for the high precipitation load associated with many TCs. Therefore, advection combined with significant inputs of suspended and dissolved matter, nutrients, increased turbidity and reduced salinity are of greater significance nearshore relative to the open ocean (Paerl *et al*., 2018, 2020; Osburn *et al.,* 2019; Yan *et al.,* 2020; Asmala *et al.,* 2021). On the time-scale of days to weeks the impacts of a TC on phytoplankton ecology are likely to be dominated by the amount of rainfall and the residence time of the receiving water body (Paerl *et al.,* 2018). Over a longer time-scale of months to years the impacts of TC on phytoplankton ecology include significant nutrient loads, increased turbidity and dramatically reduced salinities from stormwater runoff leading to both short- and long-term impacts on phytoplankton and biogeochemical cycling (Mallin *et al.,* 1993; Paerl *et al.,* 2001, 2018; Peierls *et al.,* 2003). Previous studies of TC impacts are primarily classic “before and after” studies (Green, 1979) providing excellent assessments of the TCs more immediate impacts. TCs also have considerable potential to cause longer term impacts on phytoplankton ecology (Hall *et al.,* 2013) but are largely speculative as seasonal progression and interannual variability limits their detection by before and after studies.

In the coastal zone, phytoplankton community composition has been reported to be impacted by TCs with increases in dinoflagellates and sometimes diatoms (Tsuchiya *et al.,* 2014; Anglès *et al.,* 2015; Paerl *et al.,* 2018). The exact nature of the phytoplankton response is reported to be dependent upon the characteristics and timing of the storm. Based on only a few locations in the northern hemisphere, it is suggested that wet storms with higher runoff may favor dinoflagellates, especially during the second half of the year (Tsuchiya *et al.,* 2014; Anglès *et al.,* 2015; Paerl *et al.,* 2018). In some cases, the rapid responses of phytoplankton taxa over periods of days (Tsuchiya *et al.,* 2014; Anglès *et al.,* 2015) suggest the most obvious impacts in the coastal zone can be due to advection (Peierls *et al.,* 2012; Hall *et al.,* 2013; Harding Jr. *et al.,* 2016).

## METHODS

In this study, we used long time series of environmental and phytoplankton observations to create a climatology (monthly long-term average (LTA) conditions) and compared them with the conditions observed after a TC. Our objective was to identify the impacts of TCs on the phytoplankton community and ecology. The approach makes it possible to assess impacts over several months that would normally be confounded by seasonal progression. Where appropriate we have also made before and after comparisons (Green, 1979). For example, TCs are more common in hotter than average years. During a long, hot summer stratification may increase and nutrients within the euphotic zone may decline below their LTA. In some locations, such as Australia, hot years also tend to be wetter than normal, producing lower salinities even before the TC develops. In these circumstances of a persistent deviation from normal conditions, the before and after comparison may be more useful in detecting TC impacts than a comparison with climatological averages.

The sites varied in the number of stations and depths sampled plus the observations varied in interval from minutes to months (Table I). Unless otherwise specified, the monthly data or monthly averages were used for analysis. The general approach was to use the long-term time series data to produce a climatology (long term monthly averages = LTA) at the site and then compare these with observations obtained before and after the TC has passed over the site. The results are generally provided both as the percent difference from the LTA in summary figures and with more conventional units reported in the supplemental figures. Post TC observations outside the climatology’s estimated 95% confidence intervals (Sokal and Rohlf, 1973) are presented as potential effects of the TC. Differences in variables before and after the TC were also assessed particularly when there was considerable interannual variation in the variables prior to the TC event reported upon.

**Table I:** General details of locations, water bodies, sample depths and sample frequency for analysis of impacts associated with hurricanes, cyclones and typhoons

Site, location or station	Type	Event	Latitude	Longitude	Depth at site (m)	Temporal coverage used in long term average	Samples used herein
Site 1. Great Barrier Reef Lagoon. Yongala National Reference Station, ~20 km from shore.	Lagoon ~350 000 km^2^	Cyclone Yasi. 02/2011.	31°40′N	64°10′W	~27	~ monthly 9/2009–7/2017	Single station, up to 4 depths. A full description of the sampling and analytical methods can be found at: https://imos.org.au/facilities/nationalmooringnetwork/moorings-documentation Data available at: https://portal.aodn.org.au/ Methods are described in Lynch *et al.* (2014). Burdekin River flow data from site 120006B at Clare from https://water-monitoring.information.qld.gov.au/mobile/basins/BURDEKIN.htm.
Site 2. Bermuda Atlantic Time Series Study (BATS)	Open ocean	Hurricane Nicole. 10/2016.	19°19′S	147°4′E	>4200	~ monthly 10/1998–12/2016	Single station up to 10 depths for pigments. A full description of the sampling and analytical methods can be found at: http://bats.bios.edu/about/cruise-information/ Methods also described in Steinberg *et al.* (Steinberg *et al.,* 2001). Data available at: http://bats.bios.edu/data/
Site 3. Hawaii Ocean Time series (HOT)	Open ocean	TS Guillermo. 8/2015	22°45′N	158°00′W	4750	~ monthly 10/1998–12/2018	Single station, many depths. A full description of the sampling and analytical methods can be found at: https://hahana.soest.hawaii.edu/HOT/protocols/protocols.html Data at: https://hahana.soest.hawaii.edu/hot/dataaccess.html Methods also described in Karl and Lucas (Karl and Lukas, 1996) and in Karl *et al.* (Karl *et al.,* 2001).
Site 4. Chesapeake Bay.	Estuary ~12 000 km^2^	Hurricane Floyd. 9/1999	37°00′ to 39°40′N	76°02′ to 76°27′W.	6–33	~ monthly 1/1987–12/2017	Data from January 1987 to December 2017 and 8 stations, multiple depths. Stations used: CB2.1 (2 depths), CB3.3C (5 depths), CB4.3C (4 depths), CB5.2 (4 depths), CB6.1 (4 depths), CB6.4 (4 depths), CB7.3E (2 depths), CB7.4 (4 depths). ‘CBP Water Quality Data can be found at: (https://www.chesapeakebay.net/what/downloads/cbp_water_quality_database_1984_present)’ and “CBP Living Resources” data at: (https://www.chesapeakebay.net/what/downloads/baywide_cbp_plankton_database). A description of the methods can also be found in Marshall *et al.,* 2005. Water quality measurements are described in US EPA 903-R-12-001 (2012) and EPA (EPA 003R 903R 12-002, 2012) The 2012 User’s Guide to Chesapeake Bay Program Biological Monitoring Data.
Site 5. Neuse River Estuary.	Estuary ~1500 km^2^	Hurricane Matthew 11/2016	~35°N	~76°45′W	2–7	~bi-monthly 1/1994–12/2016	11 stations, 2 depths. A full description of the sampling and analytical methods can be found at: https://portal.secoora.org/?#metadata/190/sensor_source/inventory Additional descriptions of the methods can be found in Paerl *et al.* (2001, 2007, 2018) Data can be found at: https://portal.secoora.org/
Site 6. Pamlico Sound.	Lagoon ~4300 km^2^	Hurricane Matthew 11/2016	~35°06′	76°19′W	2–7	~bi-monthly 10/99–11/2017	9 stations, 2 depths. A full description of the sampling and analytical methods can be found at: https://portal.secoora.org/?#metadata/190/sensor_source/inventory Additional descriptions of the methods can be found in Paerl *et al.* (2001, 2007, 2018) Data can be found at: https://portal.secoora.org/
Site 7. Port Aransas, Texas. Guadalupe, Mission-Aransas and Nueces estuaries.	Estuary & Lagoon at connection to Gulf	TS Bill 6/2015	27°50′N	97°03′W	1–3	Every 20 minutes	One station, ~4 m. Details of the data and methods can be found in Anglès *et al.* (2015) and Fiorendino *et al.* (2021). Additional description of the sampling methods can be found at the centralized data management office for the National Estuarine Research Reserve System: https://cdmo.baruch.sc.edu/request-manuals/ and data are available at: https://cdmo.baruch.sc.edu/dges/ Image data are found at https://toast.tamu.edu/timeline?dataset=PortAransas
Site 8. Hong Kong. Pearl River Estuary and nearby coastal waters.	Coastal waters	Typhoon Sam 8/1999	~22°2′N	~114°.1′E	14–34	~ monthly 1986–2004.	10 stations (NM1, NM3, SM3, SM6, SM17, SM19, MM4, MM14, MM16, MM17) *surface samples only*. Data courtesy of the Hong Kong Environmental Protection Department (https://www.epd.gov.hk/epd/english/top.html). Details in HKEPD (2012) sampling and analysis after Yin (2002).

A range of sites that both experience TCs and have a long time series of relevant observations that included measures of phytoplankton biota or biomass such as chl*a*, other pigments including zeaxanthin (Zea), divinyl chl*a* (DV chl*a*), 19′-Hexanoyloxyfucoxanthin (19-Hex), Fucoxanthin (Fuco), 19′-Butanoyloxyfucoxanthin (19-But), all identified by HPLC (Pinckney *et al.,* 2001; Schlüter *et al.,* 2006), and also including the enumeration of taxa identified to a defined group, genera or species. Where many taxa or pigments were measured, only the dominant taxa or pigment data are presented. Where sites had experienced more than one potential TC event then additional event selection criteria included greatest wind speed (e.g. category 1, or greater, TCs), greater local precipitation and runoff. Where possible, a consistent set of variables are presented for each location that include physical changes (e.g. temperature) followed by chemical (e.g. salinity and nutrients), other water quality measures (e.g. turbidity), and conclude with phytoplankton biomass as pigments and or the abundance of taxa.

Limited statistical comparisons were made, although some data sets lent themselves to comparison by analysis of variance (ANOVA; Sokal and Rohlf, 1973) and the data were log transformed to improve the statistical distribution (Tett, 1973). At open ocean sites, where numerous depths were sampled (e.g. Bermuda Atlantic Time Series [BATS], Hawaiian Ocean Time [HOT] series), the data were binned by depth. For example, at the HOT series the data were binned 0–25, 25–50, 50–100, 100–150, 150–200 m and the multiple sampling within the bins made it feasible to test the null hypothesis that the post TC conditions were equal to the LTA for each bin.

The results commence with a full description from one site in the Australian Great Barrier Reef Lagoon. The remaining sites are presented in summary form of 1 or 2 figures in the main body of the paper followed by a description of the results that may cite the details contained in supplemental figures. There three sections for each site: (i) an introduction to the site and event, (ii) a summary of the major results, (iii) a more detailed account of the results.

## RESULTS

### Site 1. The Great Barrier Reef Lagoon, Australia

**Fig. 1:**
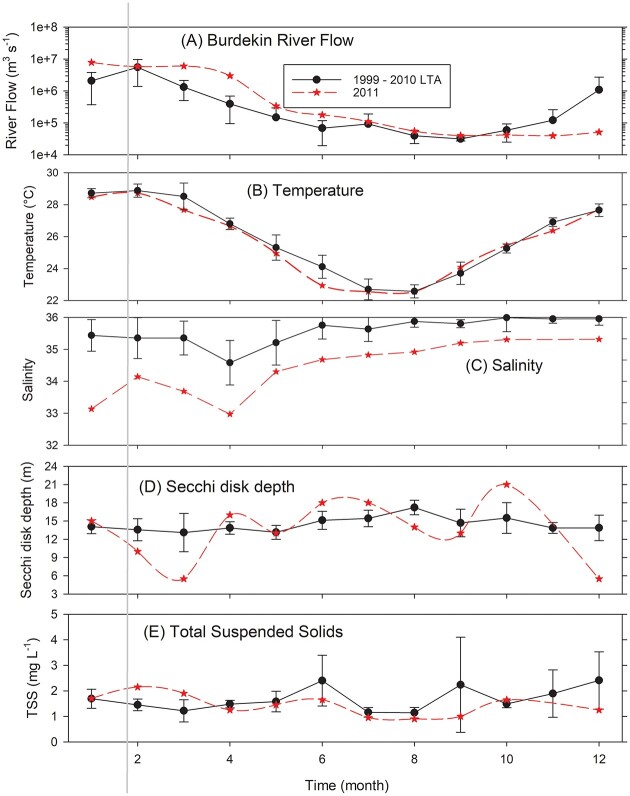
Cyclone Yasi passed over the Yongala site prior to the February 2011 sampling event. (**A**) Burdekin River flow data. Remaining panels (**B**–**E**) are based on the LTA of 168 ~monthly observations from 2009 to 2017 relative to those in 2011 at the Yongala site in the Great Barrier Reef Lagoon (19°18.5S, 147°37.1E). Where shown error bars are 95% confidence intervals. (B) Depth averaged (0–27 m) temperature and (C) salinity by month. (D) Secchi disk depths. (E) Total suspended solids. Solid vertical gray line indicates post cyclone sampling in 2011.

The Great Barrier Reef of Australia is one of the largest contiguous coral reef ecosystems in the world with 3200 individual reefs stretching over 2300 km creating a large (~200 000 km^2^) complex lagoonal habitat with terrestrial inputs of freshwater, nutrients and suspended solids that shape the pelagic ecology of this ecosystem (Brodie *et al.,* 2012). TC Yasi, one of the most powerful cyclones (TC5) ever recorded to make landfall in Australia, passed across the Great Barrier Reef on 3 February 2011 very close to the Australian Integrated Marine Observing System’s (Lynch *et al.,* 2014) National Reference Station (Yongala) in the Great Barrier Reef Lagoon where there are 4 depths sampled monthly from the surface to near the bottom at 27 m (Table I). TC Yasi was spawned during a strong La Nina event with greater than average summer rainfall across much of the eastern coast of Australia (Imielska, 2011). TC Yasi dropped 50–300 mm more rain along greater than 600 km of coastline. The rainfall in the largest catchment in NE Australia flows into Burdekin River was at historic highs with January, March and April well above the 10 y mean (Fig. 1A).

#### The Great Barrier Reef Lagoon summary results

**Fig. 2:**
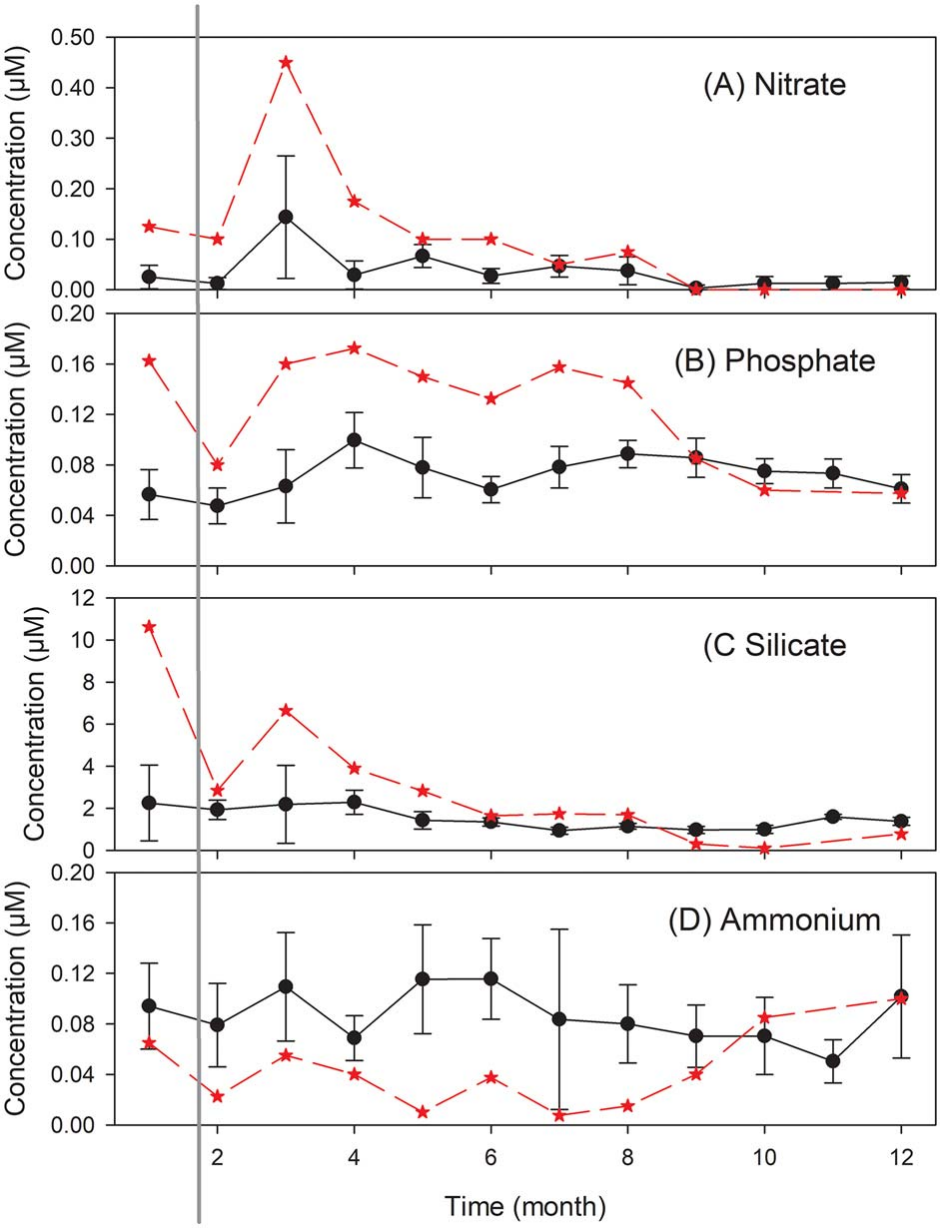
Cyclone Yasi passed over the Yongala site (19°18.5S, 147°37.1E) in the Great Barrier Reef Lagoon prior to the February 2011 sampling event. Samples were collected ~monthly from 2009 to 2017 from 4 depths. Long term depth-averaged mean monthly value (●). Monthly depth averaged values for 2011 (★). Where shown error bars are 95% confidence intervals. (**A**) Nitrate + nitrite, (**B**) Phosphate, (**C**) Silicate, (**D**) Ammonium. Solid vertical gray line indicates post cyclone sampling in 2011.

**Fig. 3:**
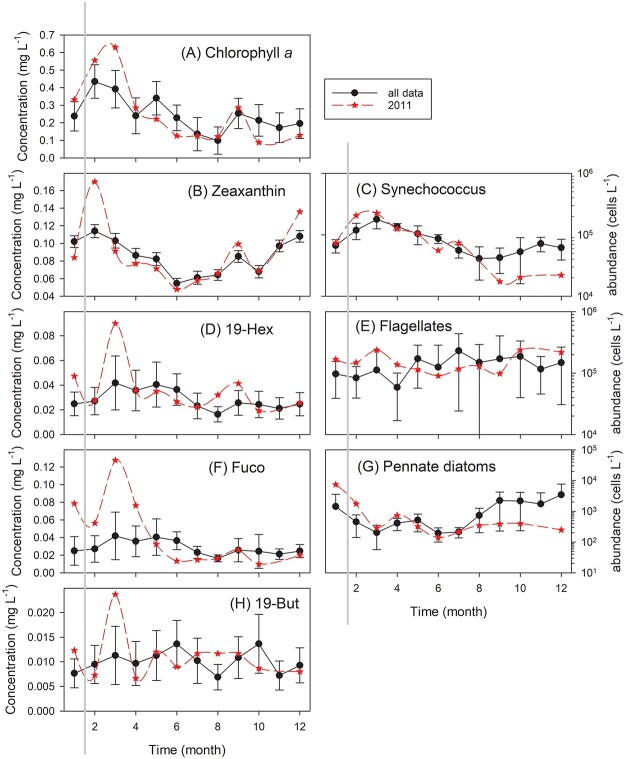
Cyclone Yasi passed over the Yongala site in the Great Barrier Reef Lagoon (19°18.5S, 147°37.1E) prior to the February 2011 sampling event. The LTA were ~monthly samples from 2009 to 2017 from multiple depths. LTA monthly value (●). Monthly values for 2011 (★). Where shown error bars are 95% confidence intervals. (**A**) Chl*a* concentration, (**B**) Zeaxanthin concentration, (**C**) *Synechococcus* abundance, (**D**) 19′-Hexanoyloxyfucoxanthin concentration, (**E**) Flagellates, (**F**) Fucoxanthin concentration, (**G**) Pennate diatom abundance, (**H**) 19′-Butanoyloxyfucoxanthin concentration. Solid vertical gray line indicates post cyclone sampling in 2011.

**Fig. 4:**
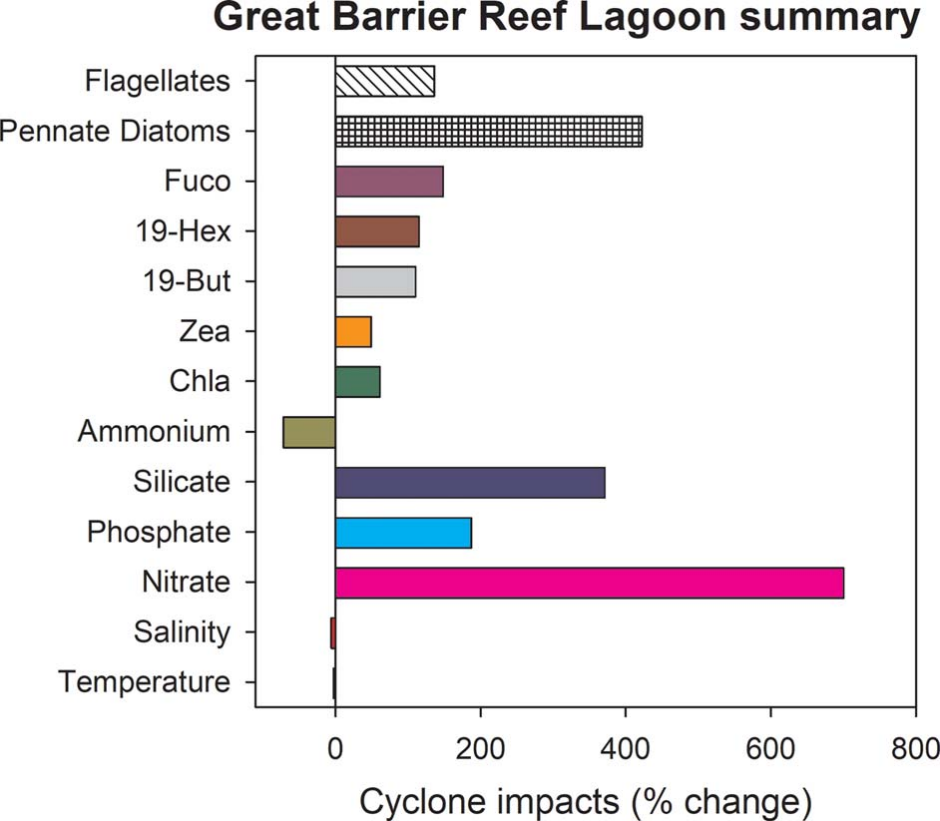
Summary of the maximum difference between the monthly LTAs and conditions observed 1–6 months after cyclone Yasi passed over the Yongala site in the Great Barrier Reef Lagoon (19°18.5S, 147°37.1E) in February 2011.

In summary, following TC Yasi the observed changes in the temperature and salinity were relatively small at −3% and −6%, respectively (Fig. 4). At this location in the Great Barrier Reef lagoon, the depth averaged increases in NO_3_ + NO_2_, SiOH_2_ and PO_4_ were 0.31, 4.46 and 0.10 μM, respectively (Fig. 2). The nutrients remained elevated for ~6 months. The observed biological responses were of an average increase of 149%; with zea rising 49%, chl*a* 61%, *Synechococcus*, 73%, 19-But 110%, 19-Hex 115%, flagellates 136%, Fucoxanthin 148% and pennate diatoms by 422% (Figs 3 and 4).

#### The Great Barrier Reef Lagoon detailed results

**Fig. 5:**
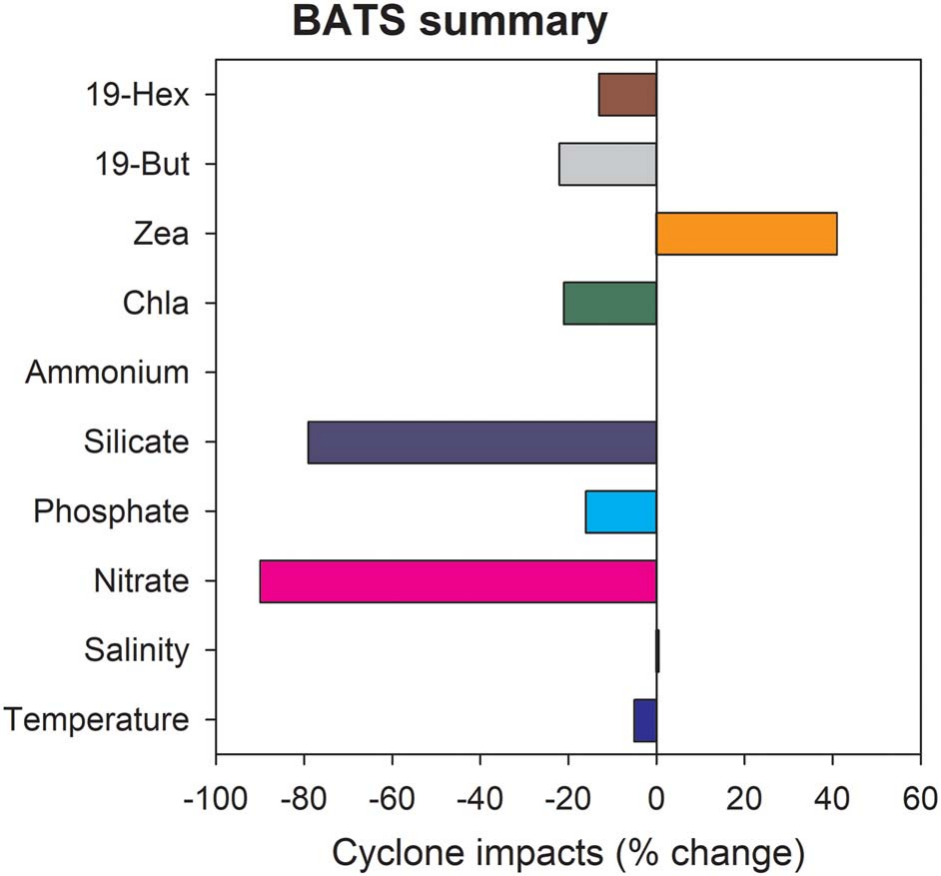
Summary of the maximum difference between the monthly LTAs and conditions observed after Hurricane Nicole passed within ~70 km of the Bermuda Atlantic Time Series (BATS, 19°19′S, 147°4′E) site in October 2016.

Following TC Yasi, the water temperatures throughout the water column were ~0.5°C lower than normal but not significantly different from the LTA. Salinities were well below the normal range throughout January to May, with a difference of ~3 in March (Fig. 1B). Secchi disk depth decreased to 5.5 m in February 2011 from the LTA of 14.5 ± 0.5 (mean ± 95% CI), while total suspended solids (TSS) remained above normal in February and March 2011 (Fig. 1C). Unfortunately, the mooring at the site did not survive the TC; hence, there are no continuous data for temperature or salinity during early 2011 (Fig. 5).

Nitrate, silicate and phosphate concentrations were outside normal ranges for January to June with the highest ever recorded concentration of silicate (30.1 μM) and phosphate (0.29 μM) from the surface sample in January 2011. The depth-averaged nutrient increases were not as extreme but still remarkable with an 800% increase in NO_3_ + NO_2_ during February 2011. Overall, available data for nitrate (*r*^2^ = 0.09, *P* < 0.001), phosphate (*r*^2^ = 0.28, *P* < 0.001) and silicate (*r*^2^ = 0.54, *P* < 0.001) were negatively correlated with salinity, suggesting a significant component of these nutrients was derived from terrestrial riverine inputs to the Great Barrier Reef Lagoon. On average, the molar nutrient ratios were (NO_3_ + NH_4_):PO_4_ ~ 1.7 (Redfield ~16) and (NO_3_ + NH_4_):SiOH_2_ ~ 13, indicating an ecosystem that is probably limited by the availability of N (Redfield, 1958).

Phytoplankton biomass (chl*a*) was elevated in January, February and reached its annual peak a month later than normal, in March 2011 (Fig. 3A, Table II). The increased biomass in February was primarily taxa containing zeaxanthin and included *Synechococcus* (Fig. 3B and C). The above average biomass from January to April was also high in Fuco (Fig. 3F) and pennate diatoms (Fig. 3G). The peak biomass in March was associated with small flagellates (Fig. 3E), presumably containing 19′-Hex (Fig. 3D), and 19′-But (Fig. 3H) the concentrations of which were also elevated. Following the cyclone, peridinin, a marker for photosynthetic dinoflagellates, was four times greater (*P* < 0.05) than the average March concentration (data not shown).

**Table II:** Changes in temperature, salinity, nutrients, pigments, abundance of taxa, water clarity and oxygen concentrations that were impacted by the passage of a tropical cyclone reported relative to the relevant long term monthly average

Site and event	Physical change		Salinity and nutrients % change					Biological % change	
	Temperature %	°C	Salinity	NO_3_ + NO_2_	PO_4_	SiOH_2_	NH_4_	Pigment concentrations or abundance of Taxa	Other
Site 1. Great Barrier Reef Lagoon. 19°19S, 147°37E Cyclone Yasi. 2011.	−3 (ND)	−0.84^a^	−6	+700	+187	+371	−72	Chla +61, Zea +49, 19But +110, 19Hex +115, Fuco +148, *Synechococcus* +73, Pennate diatoms +422, Flagellates +136	Secchi disk depth −62
Site 2. Bermuda Atlantic Time Series Study (BATS). 31.6°N, 64.2°W Hurricane Nicole. 2016.	−5	−1.2^a^	+0.5	−90	−16 (ND)	−79%		Chla −21 19-Hex −13 Chlb −30 19-But −22 Zea +41	
Site 3. Hawaii Ocean Time-series (HOT). 22.5°N, 158°W TS Guillermo. 2015. Average changes between 100 and 150 m depth.	−11	−2.5	−4	109	18	38		Chl*a* +12, 19But +29, 19Hex +6, Peridinin −54, Chl*b* +27, Fuco −2, Zea +8 Prochlorococcus +6, *Synechococcus* −29, picoeukaryotes+15	
Site 4. Chesapeake Bay. ~ 37.2°N, 76.1°W. Hurricane Floyd. 1999.	All STN −6 STN CB6.4 −11.8 STN CB7.4 −2.0	All STN −1.52	+3 above LTA, −7% from previous month in 1999.	+62	−38	−45	−32 +20^a^ +19^a^	Chla +38, −11^a^, −7^b^, −38^3^ *Cerataulina pelagica* −98 *Leptocylindrus minimus* −25 *Cylindrotheca closterium* +6 *Pseudo-nitzschia pungens* −79 *Dactyliosolen fragilissimus* +66 *Skeletonema costatum* +330 *Thalassionema nitzschioides* +11 *Gymnodinium* +31 *Prorocentrum minimum* −27 *Katodinium rotundatum* +112 Green cells −34 Micro-phytoflagellates −84 Cryptomonas +11	TSSs +48 Secchi disk depth −17 Dissolved oxygen +15 Extinction coefficient −5 (+36^a^)
Site 5. Neuse River Estuary. ~35°N, 76.7°W Hurricane Matthew. 2016.	+4	+0.78	−39 STN 20 −99% STN 180 −45%	+4 (ND)	+70	−53	+26 +114^a^ STN 30 −62 STN 120 +257^a^	Chla −49, Chla +30^b^, Fuco −64, Peridinin −10, Zea −51, 19Hex −96, Allo −45, Chlb −44.	Dissolved oxygen −25 Secchi disk depth −43 Extinction coefficient +64
Site 6. Pamlico Sound. ~35.1°N 76.3°W. Hurricane Matthew. 2016.	−9	−1.68	−45	+216^a^	−54	−29	+34^a^	Chl*a* +2, Chl*b* +62, Alloxanthin +42, Lutein −65, Zea −84, Viola −69, Antheraxanthin −35.	Dissolved oxygen +11 Secchi disk depth −25 Extinction coefficient +34
Site 7. Port Aransas, Texas. Guadalupe, Mission-Aransas and Nueces estuaries. 27°5 N, 97°3 W. TS Bill 2015.	−0.4 (ND)	−0.13	−14 −20^a^	−48 −77^a^	−63		−31	Chl*a* −21, Chl*a* +48^a^	DO +1.2 DO −7.2^a^ DO −11.5^b^ NTU +179 NTU +421^a^
Site 8. Hong Kong. Pearl River Estuary. ~22.3°N 114.2°E. Surface data averaged across all sites except temperature. TC Sam 1999.	Overall sites 0 (ND) NM (near PRE) -4 SM (mid) -2 MM (far) +6	Overall sites 0 NM (near PRE) −1.17 SM (mid) −0.50 MM (far) +1.67	−9	+35	+54	+43	+67	Diatoms +94 Dinoflagellates+211 Other taxa +264	DO+7 Turbidity+43 Suspended solids +50

^a^More than 1 month past event.

^b^More than 2 months past event.

Results are reported here are outside the 95% confidence interval from the LTA unless specified as not different (ND). Data details are specified in figures for each location and parameter. Temperature results are reported in °C and as the percent (%) difference from the LTA. Other results are reported only as the percent (%) difference from the long-term monthly average.

### Site 2. Bermuda Atlantic Time Series

The BATS site is located ~75 km SE of Bermuda at ~31°40′N 64°10′ in about 4500 m of water, providing samples representative of the North Atlantic subtropics (Phillips and Joyce, 2007). On 13 October 2016, category 3 Hurricane Nicole passed within 200 km of the BATS station with winds gusts as high as 219 km/h and rainfall of 172 mm, becoming one strongest and wettest recorded storms to impact Bermuda. Regular sampling at the BATS station was undertaken shortly after from 16 to 21 October 2016.

#### BATS summary results

During the summer of 2016 surface waters were modestly warmer (Supplementary Fig. S1A) and by September considerably saltier than normal at BATS (Supplementary Fig. S1B). These became 5% colder and closer to normal salinity with the passage of Hurricane Nicole (Supplementary Fig. S1A and B, respectively). The nutricline was very deep during the summer of 2016 and nutrients in the euphotic zone remained well below the LTA (Fig. 5) from March to December 2016. In September 2016 NO_3_ + NO_2_ were reported as zero from the surface to 100 m. The depth averaged concentration of the major marker pigments; chl*a*, 19-Hex, 19-But, chlorophyll *b*, remained below the LTA from September to December 2016 (Supplementary Fig. S2A–D, respectively), except for zeaxanthin (Supplementary Fig. S2E).

#### BATS detailed results

The BATS surface waters were at, or above, normal temperatures from January to September 2016, only falling below normal after October (Supplementary Fig. S1A). In September 2016 salinity at 10 m had risen significantly (0.35) above the LTA, then fell 0.20 post TC although still remained above the LTA until November (Supplementary Fig. S1B). Surface stratification (0–200 m) was greater than normal prior to Hurricane Nicole and, in association with more rapid than normal surface cooling, both surface temperatures and stratification were below normal by December 2016 (Supplementary Fig. S1D). The density gradient between 0 and 200 m was 0.1 kg m^−3^ greater than the LTA in August 2016, but by December this was 0.6 kg m^−3^ less than the LTA (Supplementary Fig. S1D), indicating a faster than normal reduction in stratification post TC.

Nutrient concentrations were generally very low throughout most of 2016 relative to the LTA. Concentrations of NO_3_ + NO_2_ were reported as zero from the surface to near the bottom of the euphotic zone during the summer of 2016 (Supplementary Fig. S1C, Table II). At 140 m across all months with data (April—December) for 2016 NO_3_ + NO_2_ averaged only 0.53 μM or 39% of the LTA (*P* < 0.001); while SiOH_2_ was 0.19 μM or 21% of the LTA (*P* < 0.001); and PO_4_ was 0.037 μM or 16% lower than the LTA (NSD). At 140 m NO_3_ + NO_2_ remained ~1 μM lower than the LTA from April 2016 until October 2016 (Supplementary Fig. S1C). Following Hurricane Nicole, NO_3_ + NO_2_ concentrations at 140 m rose to 0.8 μM by December 2016 (Supplementary Fig. S1C) suggesting increased vertical mixing. The rapid cooling of the surface post TC was also associated with a deepening of the thermocline as the surface mixed-layer depth increased rapidly from ~30 m in September 2016 to ~90 m November 2016 (Supplementary Fig. S1E).

From September to December 2016 the depth averaged (0–250 m) chl*a* was lower than the LTA although it was closest in October (Supplementary Fig. S2A). The main marker pigments were: 19 Hex > chlorophyll *b* > zea > chlorophylls c_1_ + c_2_ > 19 but > chlorophyll c_3_ > fuco > diadinoxanthin > prasinoxanthin > alloxanthin > peridinin > diatoxanthin > lutein. All of the top five marker pigments with the exception of zea were below LTA values from September to December 2016 (Supplementary Fig. S1B–E, respectively). Only zea showed a likely response to Hurricane Nicole, rising to be 141% of its depth averaged LTA and outside the LTA’s 95% confidence intervals post TC in November 2016 (Supplementary Fig. S1E) when the zea concentrations were greater than the LTA at all depths (0–120 m) and ~twice the LTA at 100 m (Supplementary Fig. S2I).

The vertical profiles of temperature, salinity, nutrients and chl*a* suggest that mixing from the surface down by Hurricane Nicole did disturb the deep chl*a* maximum at 100 m (Supplementary Fig. S2F). The TC did appear erode the thermocline injecting more nutrients from below and growing the strong chl*a* maximum (chl*a*_max_) of 0.300 μg kg^−1^ at 100 m. This chl*a*_max_ was both greater than normal and still 20 m deeper (Supplementary Fig. S2F) post TC. The latter possibly reflecting the long summer of low nutrients in the surface mixed layer (Supplementary Fig. S1C). The deep chl*a*_max_ persisted into November (Supplementary Fig. S2G) but was largely eroded by December 2016. Following the passage of Hurricane Nicole, the chl*a* concentrations nearer to the surface in November (0–80 m, Supplementary Fig. S2G) and in December (0–100 m Supplementary Fig. S2H) remained less the LTA.

Typically, between October and December there is a gradual increase in depth-averaged chl*a* (Supplementary Fig. S2A) that continues until the annual peak is reached in February. This increase is largely associated with rising chl*a* concentrations within the surface mixed layer (~0–60 m, Supplementary Fig. S2F–H). During late 2016 the amount, and rate of increase in, chl*a* within the surface mixed layer was significantly less than normal (Supplementary Fig. S2F–H). The results indicate that although Hurricane Nicole increased the standing stock of phytoplankton in late 2016 the 0–250 m average annual phytoplankton biomass in 2016 was only 0.96 μg kg^−1^ relative the LTA of 1.12 μg kg^−1^. Of the major phytoplankton taxa present at BATS, *Prochlorococcus*, Haptophytes (possibly coccolithophores), Pelagophytes and cyanobacteria increased modestly from their September lows and appeared to potentially benefit from the conditions associated with the passing of the TC.

### Site 3. Hawaiian Ocean Time series, USA

The HOT series is obtained ~100 km north of Oahu, Hawaii within a 10 km radius of 22°45′ and 158°W in ~4800 m of water and sampling the North Pacific subtropical gyre (Karl and Lukas, 1996). Since 1949 there have been ~66 major storms affecting Hawaii. Thirty of these occurred in August. In association with a strong ENSO event, the 2015 central Pacific Hurricane season was the most active on record with 16 TCs. In August 2015, for the first time on record, there were three category four cyclones between 140 and 180°W. Only Guillermo came within ~100 km of HOT on 7 August 2015 after weakening from category 2 to a Tropical Storm (TS).

#### HOT summary results

**Fig. 6:**
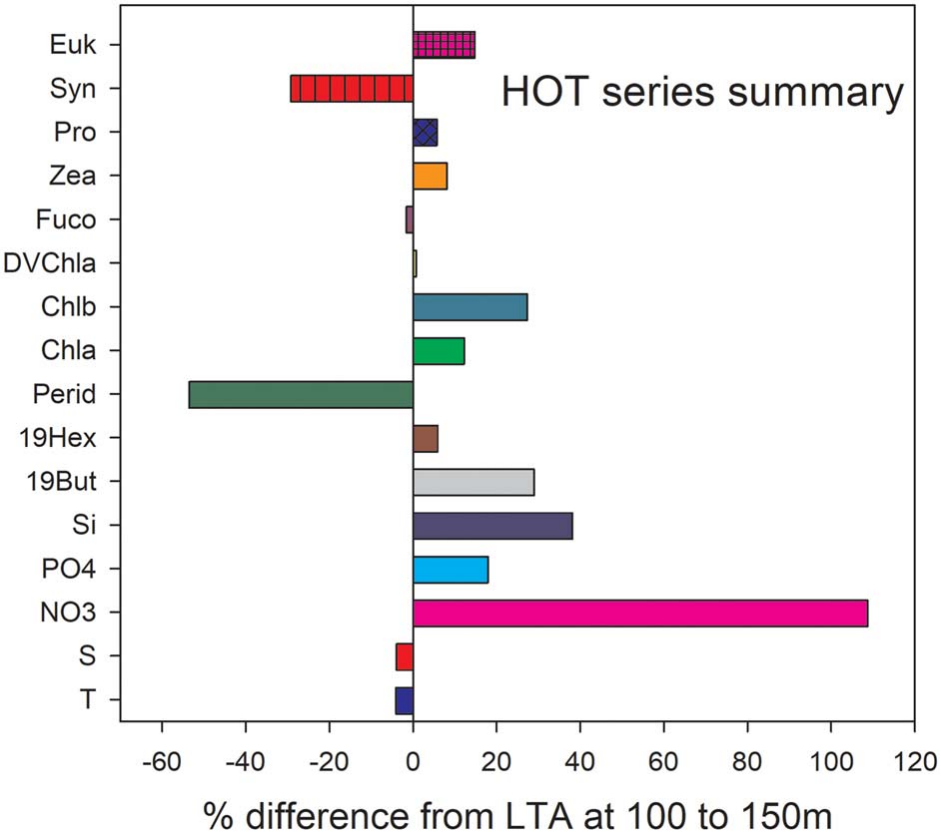
Summary of the differences between the monthly LTAs and conditions observed ~2 months after TS Guillermo passed within 100 km of the HOT series at 22°45′N & 158°00′W August 2015.

The surface mixed layer was slightly deeper and warmer in August 2015 post TS than it had been in July, while at depths from 75 to 300 m temperatures fell markedly (Supplementary Fig. S3A). Following the passage of TS Guillermo in August, the maximal differences from the LTA conditions tended to be found at depths between 100 and 200 m (Supplementary Fig. S3E) and some persisted for ~2 months (Supplementary Fig. S3A–C). Between 100 and 150 m the temperature and salinity were 4% below the LTA by October 2015 (Fig. 6). In August 2015, just post TS Guillermo, nutrients were well above average at depths of 100–200 m with NO_3_ up 108%, PO_4_ up 18% and Si +38% (Fig. 6). Also at depths between 100 and 200 m eukaryote taxa such as pelagophytes (chl*a*, 19-Hex and 19-But), *Prochlorococcus* (cell counts and DV chl*a*, zeaxanthin) were all well above the LTA. Other taxa such as photosynthetic dinoflagellates (peridinin) and *Synechococcus* (cell counts) were present in lower-than-normal abundances (Fig. 6).

#### HOT detailed results

The temperature of the upper 200 m of the ocean at HOT averaged 0.37°C warmer than the LTA through June and July 2015. At depths >50 the oscillations in monthly temperatures suggested some mesoscale variation throughout spring and early summer; possibly due to passing eddies (Supplementary Fig. S3A). The thermocline deepened from ~37 to ~44 m following the passage of TS Guillermo. After the storm passed the difference in temperature from the LTA was largely a function of depth with deeper waters colder than LTA (Supplementary Fig. S3E), suggesting an uplifting of isotherms. At the depth range of 100–150 m temperatures fell ~2.5°C below the LTA following the TS (Supplementary Fig. S3A).

During 2015 at all depths from 0 to 200 m the salinity was also generally above the LTA from February to July. Following TS Guillermo salinity remained above the LTA at depths from 0 to 100 m while between 100 and 200 m it fell an average of 0.27 (Supplementary Fig. S2B). Generally, there was an August salinity maximum near 125 m but in August 2015 salinities were elevated between 0 and 125 m relative to the LTA (Supplementary Fig. S3E).

The long-term depth averaged (0–200 m) nutrient concentrations for August were: 0.6 μM for NO_3_, 0.11 μM for PO_4_ and 1.68 μM for Si. These N:P:Si ratios of 5:1:15 suggest N is likely to be the limiting nutrient during late summer. Following TS Guillermo, NO_3_ was elevated above the LTA at all depths (Supplementary Fig. S2E) especially between 100 and 150 m (Supplementary Fig. S3C and E) where is increased from 0.59 to 1.61 μM. Both Si and PO_4_ were also elevated 50–100% above the LTA at depths >100 m post TS Guillermo (Supplementary Fig. S3E).

During the summer of 2015 and several days after the passage of TS Guillermo, the pigments detected by HPLC were largely below their LTAs. For example, from 0 to 200 m in August 2015 chl*a* averaged 36 ± 17% lower than normal (Supplementary Fig. S3E). Pigment data for September was not available but by October 2015 chl*a* had risen to be up 30%, or 27 μg m^−3^, greater than the LTA between 100 and 150 m (Supplementary Fig. S1D). The increase was even greater between 150 and 200 m with chl*a* increasing 115%, or 41 μg m^−3^, relative to the LTA. By October 2015 many deep-living taxa appeared to have responded to the TS with increases in eukaryote taxa such as pelagophytes (more chl*a*, 19-Hex and 19-But), *Prochlorococcus* (cell counts and DV chl*a*, zeaxanthin) were well above the LTA (Table II). Other taxa such as photosynthetic dinoflagellates (peridinin, 54% below LTA) and *Synechococcus* (cell counts, 29% below LTA) were present in lower-than-normal abundances at depths 100–150 m (Fig. 6).

### Site 4. Chesapeake Bay, USA

Chesapeake Bay is a very large estuary on mid-Atlantic coast of Virginia and Maryland, USA. It is ~320 km long and from 4.5 to 48 km wide. It has ~19 000 km of shoreline, a surface area of ~12 000 km^2^, an average depth 6.4 m and a maximum of 53 m. This region of the USA experiences many TCs primarily from May through to November with September receiving >40%. For the purposes of this study, Hurricane Floyd, one of the most extreme events in terms of precipitation and impact on salinity between 1/1987 and 12/2017, was selected. Some of the regional records for 24 h rainfall were set as Hurricane Floyd traveled up the east coast of the USA in September 1999. For example, 0.31 m in 24 h in the adjacent State of Maryland at Chestertown on the 15 September 1999. Hurricane Floyd resulted in 57 fatalities, most rivers exceeded their 500 y flood levels and the surface salinity at the mouth of Chesapeake Bay (CB Station 8.1) fell from ~24 in August to ~18 in September (the largest monthly decline outside of spring runoff from 1987 to 2017). Others have reported on the phytoplankton of the Chesapeake (Marshall and Alden, 1997; Marshall *et al.,* 2005, 2006) including impacts of extreme events such as Hurricane Isabel (Miller *et al.,* 2006) and hydrodynamic modeling of Hurricane Isabel and Floyd (Cho *et al.,* 2012).

For this study, water quality data were restricted to the “Tidal Mainstem” monitoring stations, primarily those with matching phytoplankton data, specifically: CB2.1 (2 depths), CB3.3C (5 depths), CB4.3C (4 depths), CB5.2 (4 depths), CB6.1 (4 depths), CB6.4 (4 depths), CB7.3E (2 depths), CB7.4 (4 depths). These stations averaged 47 km apart roughly down the middle of the estuary with CB2.1 at the riverine end and CB7.4 at the seaward end (EPA, 2012). Water quality parameters were normally recorded at least monthly at 2–5 depths per station and phytoplankton at 2 depths per station. Over the time period analyzed for the LTA (1987–2017) the number of observations of each variable was: chl*a* (*n* = 14 724), dissolved oxygen (*n* = 58 863), *K*_d_ (*n* = 2560), NH_4_ (*n* = 14 846), NO_2_ + NO_3_ (*n* = 14 804), PO_4_ (*n* = 14 699), salinity (*n* = 58 999), Secchi disk depth (*n* = 4287), silicate (*n* = 13 044), sigma_t_ (*n* = 58 977), TSSs (*n* = 14 937), temperature (*n* = 59 011).

#### Chesapeake Bay summary results

**Fig. 7:**
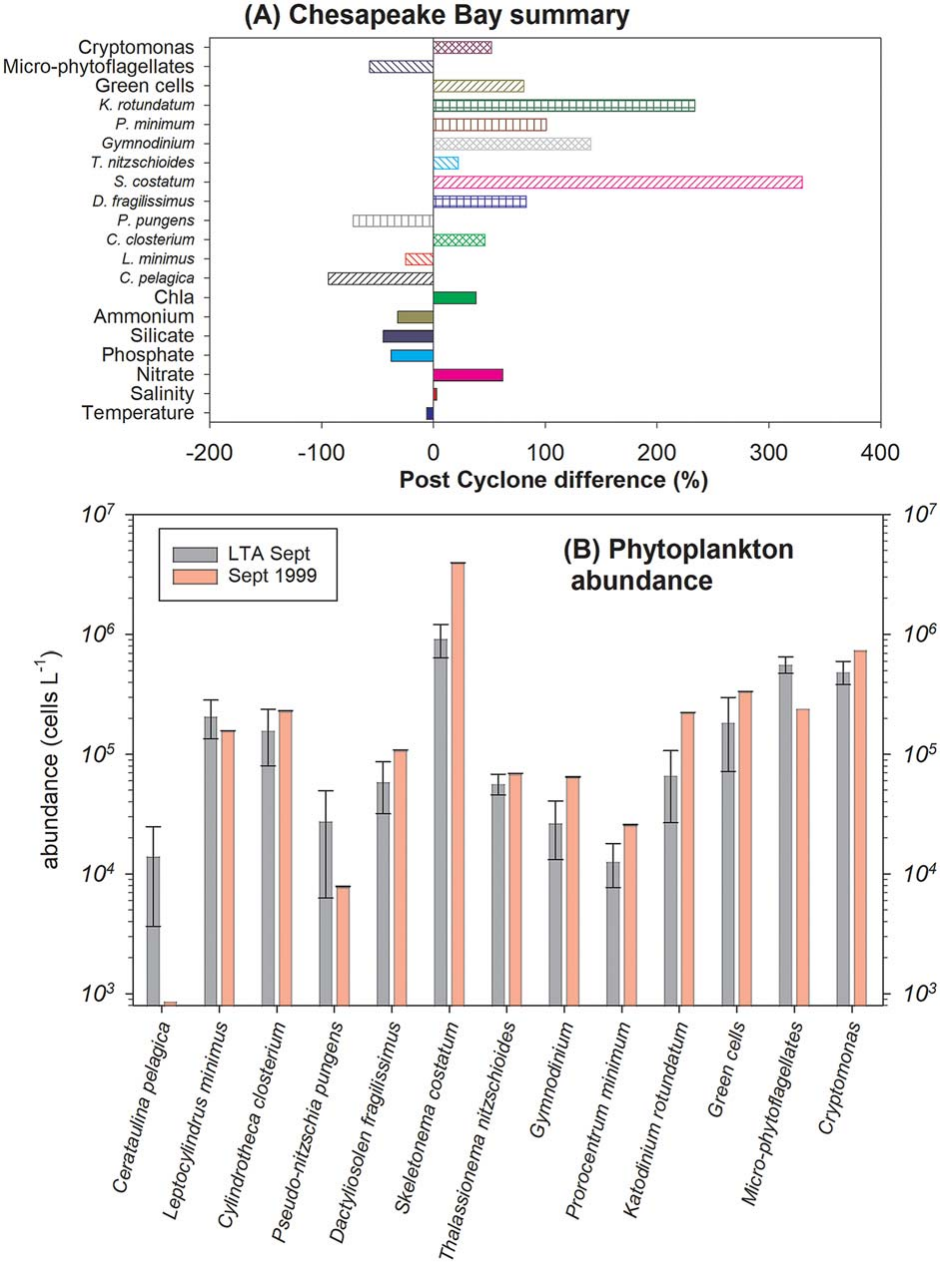
Chesapeake Bay water quality data vertically and horizontally averaged from the “Tidal Mainstem” monitoring stations (CB2.1, CB3.3C, CB4.3C, CB5.2, CB6.1, CB6.4, CB7.3E, CB7.4 after Hurricane Floyd relative to the 1987–2017 long term average (LTA). (**A**) The percent difference between observations in September 1999 and the LTA values. (**B**) The LTA ± 95% confidence intervals (gray bar) for most common phytoplankton compared with observations in September 1999 (red bar).

In September 1999 following Hurricane Floyd, the depth-averaged water temperatures for the eight mainstream stations along ~272 km of the central axis of Chesapeake Bay fell 6% (−1.52°C) below the LTA (Fig. 7). The eight station mean depth-averaged salinity was 8.6% above the LTA throughout all of 1999 (Supplementary Fig. S4) but then fell to only 2% (0.53) above TLA post Hurricane Floyd (Fig. 7). In September 1999, the TSS averaged 47% greater than LTA (Table II). During 1999 the Secchi disk depth showed negative covariance with TSS and fell 17% below the LTA following the passage of Hurricane Floyd. The average extinction coefficient (*K*_d_) was not greater than the LTA except in October, a month after Hurricane Floyd (Supplementary Fig. S4E). In general, nutrient concentrations in Chesapeake Bay were lower than normal during 1999 and only NO_2_ + NO_3_ exceeded the LTA rising from 0.03 μg L^−1^ in August to 0.14 μg L^−1^ (+62%) 1-month post TC Floyd.

From January to June 1999, the station and depth averaged chl*a* concentrations were predominantly below their LTAs with September being the only month in 1999 when the vertically and horizontally averaged chl*a* concentration in Chesapeake Bay (*n* = 29) was greater than the LTA, up 38% (Fig. 7A). Responses by phytoplankton taxa to Hurricane Floyd across all stations and depths for the 13 most abundant “taxa” (6 diatoms, 3 dinoflagellates and several broader groups such as *Cryptomonas*, micro-flagellates and chlorophyte cells) were mixed (Fig. 7B). In 1999, those taxa outside the long-term 95% CI for September (Fig. 7) were: *Cerataulina pelagica* (−98%), *Pseudo-nitzschia pungens* (−79%), *Dactyliosolen fragiissimus* (+66%), *Skeletonema costatum* (+330%), *Katodinium rotundatum* (+112%), micro-flagellates (−85%). A large rise in chl*a* at station CB7.4 was largely due to the 497% increase in *S. costatum* to ~12 × 10^6^ cells L^−1^ at that station (data not shown).

#### Chesapeake Bay detailed results

The mean monthly temperature rose from 3.7 in February to 25.6 in August and in 1999 it was slightly greater than the LTA before Hurricane Floyd and slightly less afterwards (Supplementary Fig. S6A). Salinity was 8.6% above the LTA throughout all of 1999 (Supplementary Fig. S4B) while in a normal hydrologic year salinity rises 0.41 between August and September but following Hurricane Floyd there was a reversal of the normal seasonal trend and a decline of 1.39 (Supplementary Fig. S4B). Only stations CB2.1 and CB7.3 fell below their LTA (Table I). Monthly mean TSS varied above and below their LTA throughout 1999 (Supplementary Fig. S4C). In September 1999, they averaged 47% greater than normal. The greatest difference from the LTA was observed at station CB7.4 where TSS were 186% greater than normal in September 1999. During 1999, the Secchi disk depth was also quite erratic and generally showed negative covariance with TSS (Supplementary Fig. S4D). The average Secchi disk depth fell 17% below the LTA following the passage of Hurricane Floyd with the largest declines of 33 and 37% at stations CB 2.1 and CB 7.4, respectively. During 1999, the average extinction coefficient (*K*_d_) was only elevated above the LTA in October (Supplementary Fig. S4E).

In general, nutrient concentrations in Chesapeake Bay were lower than normal during 1999 (Supplementary Fig. S5A–D). In 1999, the mean concentration of NO_2_ + NO_3_ remained below the LTA until after Hurricane Floyd, when it rose to be 62% above (Supplementary Fig. S5A). Large increases in [NO_2_ + NO_3_] were observed at stations CB2.1 and CB3.3C with all other stations showing a decrease. Mean NH_4_, PO_4_ and Si all remained less than the LTA in September 1999 (Supplementary Fig. S5B–D). The data suggest that post Hurricane Floyd both NH_4_ and Si showed more rapid than normal seasonal declines in mean concentrations (Supplementary Fig. S5C and D, respectively).

Like nutrients, the chl*a* concentrations during 1999 were predominantly below the LTA from January until July (Supplementary Fig. S6A). September was the only month in 1999 when the vertically and horizontally averaged chl*a* concentration in Chesapeake Bay (*n* = 29) exceeded the LTA with an estuary wide increase in September 1999 of +38%. At the more riverine end of the estuary (station CB2.1) chl*a* concentrations fell from Aug to Sept from 12.3 to 2.7 μg L^−1^ to be some 59% below the LTA (Supplementary Fig. S6B). Conversely at the oceanic end of the estuary they increased from 5.1 μg L^−1^ in August to 13.4 μg L^−1^ in September to be 164% above the LTA (Supplementary Fig. S6C).

### Site 5. Neuse River Estuary, North Carolina, USA

The Neuse River Estuary (NRE) is a drowned river valley ~70 km long with an average depth of 3.5 m. The Neuse River has an average annual flow of 4.7 × 10^9^ m^3^ and provides the largest share of freshwater that enters Pamlico Sound. The NRE is dominated by wind driven mixing, followed by tidal mixing and is frequently stratified during summer months, with extensive bottom water hypoxia (Luettich *et al.,* 2002). North Carolina is ranked fourth for hurricanes making landfall in the USA, after Florida, Texas and Louisiana; having experienced 35 landfalls between 1996 and 2017. About 60% of these occurred in August and September. Hurricane (TC3) Matthew, which made landfall 8 October 2016, produced record rainfall in many localities, the Neuse River peaked and overflowed reaching a record height of 8.6 m at Kinston. The NRE-Pamlico Sound is relatively shallow with an average depth <5 m, with a surface area of ~5790 km^2^. It is bounded by the Outer Banks, greatly restricting water exchange with the coastal Atlantic Ocean, creating lagoonal conditions (Paerl *et al.,* 2001). Results for the NRE are separated herein from those of Pamlico Sound as examples with different average salinities (7.6 vs 19.0, respectively), residence times (7–200 d vs 200–>400 d, respectively) and different phytoplankton communities (Mallin, 1994; Paerl *et al.,* 2007).

#### The Neuse River Estuary summary results

**Fig. 8:**
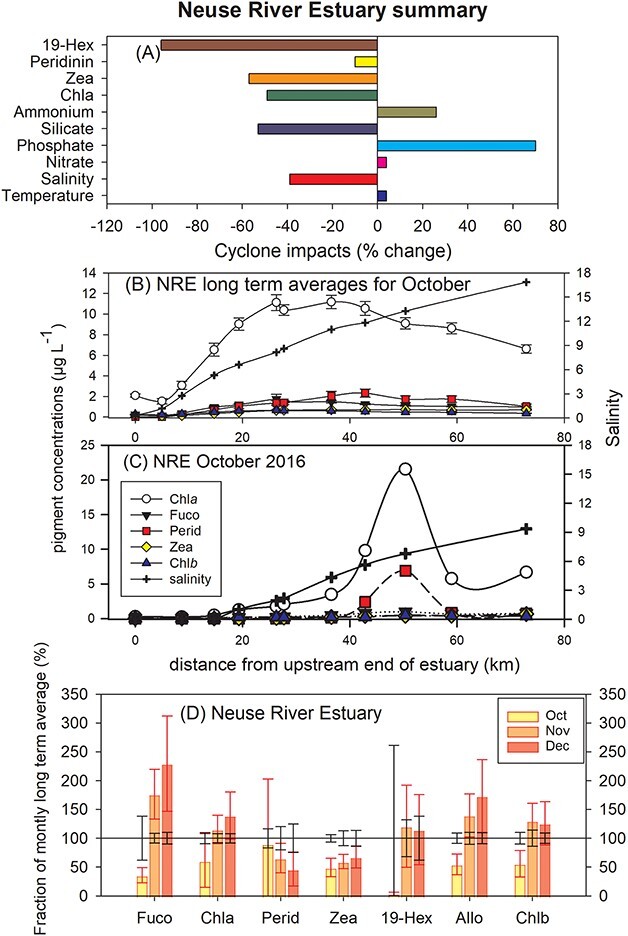
NRE summary. (**A**) The 1994–2016 LTA pigment concentrations for all stations and depths during the month of October. (**B**) The LTA depth averaged pigment concentrations and salinities for October plotted against distance the riverine end of the NRE (35.21°N, 77.12°W). (**C**) Same as (B) except October 2016 only. (**D**) Line at 100% is the LTA pigment concentrations for all stations and depths during October, November and December ±95% confidence intervals. Bars are the percentage of the LTA ± 95% confidence intervals observed in the same months in 2016.

Following Hurricane Matthew’s passing nearby the NRE in September, 2016 temperatures remained 4% warmer than normal (Supplementary Fig. S7A and Fig. 8A). Salinity, however, fell from 6.9 to 3.3 to be 39% below the LTA Fig. 8B, (Supplementary Fig. S7B). Nitrate, phosphate and ammonium were all above normal in September while silicate fell to only 53% of the LTA. All pigments declined ranging from −10% for peridinin to −96% for 19-Hex, reflecting the high freshwater flushing of the estuary. Secchi disk depth declined 43% and DO was down 25% (Table II).

Prior to the cyclone there was a broad maximum of chl*a* and other pigments distributed from ~25 to 45 km from the river mouth (Fig. 8B). Following Hurricane Matthew, estuary transect sampling revealed a distinct peak in abundance around 50 km down the estuary. This peak was heavily dominated by peridinin (Fig. 8C). Vertical profile sampling revealed the photosynthetic dinoflagellates were restricted to a layer below the fresher waters at the surface and at the top of a well-defined salt wedge.

The time series of observations indicates that phytoplankton (chl*a*) recovered to normal abundances within 1 month of the cyclone (Fig. 8D). The pigments 19-Hex, Alloxanthin and chl*b* all recovered to normal within one month, while fucoxanthin rose above the normal range in November and December 2016. The marker pigment zeaxanthin was the slowest to recover and remained low through December.

#### The Neuse River Estuary detailed results

From June to November 2016, the NRE was generally warmer than normal and following the passage of Hurricane Matthew the temperature of the NRE was closer to, but still above, the LTA (Supplementary Fig. S7A, Table II). Salinity was already less than normal before the hurricane, due to an unusually rainy summer season (Paerl *et al.,* 2018), but fell substantially to be 39% below the seasonal norm post hurricane with recovery taking longer than 3 months (Supplementary Fig. S7B). Along the estuary, the largest difference in salinity between the LTAs and November 2016 was at the most seaward station where it was ~9 relative to the LTA of nearly 18 (Fig. 8B and C, respectively). The largest percentage change occurred near the upstream riverine end of the estuary (NRE Station 20) where salinity fell 99% from 2.8 to 0.04. Dissolved oxygen was temporarily depleted by 25% to 4.9 mg L^−1^ following the passage of the hurricane, due to high organic matter loading (Asmala *et al.,* 2021; Osburn *et al.,* 2019) but recovered within a month (Supplementary Fig. S7C). Secchi disk depth fell −43% to 0.66 m and the extinction coefficient rose +64%; both impacted by the hurricane with hydrologic recovery times of three, or more, months (Fig. 7E and F, respectively).

Averaged across the NRE the concentrations of nitrate + nitrite did not show a strong response to Hurricane Matthew (Supplementary Fig. S8A). In contrast, ammonium rose significantly in the 2 months following the hurricane reaching 113 μg L^−1^ or 114% above seasonal norms by November 2016 (Supplementary Fig. S8B). This increase was attributed to the elevated organic matter load from Matthew’s floodwater (Osburn *et al.,* 2019). While the average NH_4_ concentration rose 70% there were declines of up to −62% at the riverine end of the estuary and a month later rises of up to 257% at the seaward end (Table I). The phosphate concentration was 70% greater than normal in October 2016 but returned to normal by November (Supplementary Fig. S8C). Silicate concentrations declined strongly in September and October 2016 falling 53% and then recovering fully by December (Supplementary Fig. S8D). Chl*a* was already below seasonal norms before the cyclone made landfall but fell even further, down 49% across the estuary in October 2016 (Supplementary Fig. S8E).

In an average October, fucoxanthin is the dominant phytoplankton marker pigment closely followed by peridinin, zeaxanthin, chl*b*, alloxathin, suggesting that the order of phytoplankton abundance was roughly diatoms > dinoflagellates > cyanobacteria > chlorophytes > cryptophytes. Following Hurricane Matthew, peridinin (photosynthetic dinoflagellates) became the dominant marker pigment in the NRE. The depth averaged chl*a* concentration along the entire estuary was normally ~7.5 μg L^−1^ and declined sharply to 4.5 μg L^−1^ following the cyclone (Fig. 8). A very strong chl*a* peak persisted at ~50 km from the riverine end of the estuary at 21 and 7 μg L^−1^ peridinin (Fig. 8). The highly motile dinoflagellates appear to preferentially survive *in situ* presumably resisting advection by due to a deeper distribution in the water column (Hall *et al.,* 2015).

In spite of being largely flushed out of the estuary by the cyclone, the diatoms were quick to recover, with fucoxanthin concentrations rising sharply in the months following Hurricane Matthew. Based on marker pigments the other taxa recovering quickly were cryptophytes, chlorophytes and haptophytes (Fig. 8D). All of these recovered to >100% of seasonal norms within 1 month of the hurricane. Other dominant taxa such as dinoflagellates and cyanobacteria remained below normal abundance past sampling in December 2016 (Fig. 8D).

### Site 6. Pamlico Sound North Carolina, USA

Pamlico Sound is the largest lagoonal estuary along the North American east coast, ~130 km long and 24–32 km wide averaging 1.7 m deep with a maximum depth of 8 m and an average water residence time of ~1 y (Paerl *et al.,* 2001, 2018). Most of the freshwater entering comes from the Neuse River but also from the Tar, Pamlico and Trent Rivers (Bales, 2003). Hurricane Matthew passed nearby as a category 1 TC.

#### Pamlico sound summary results

**Fig. 9:**
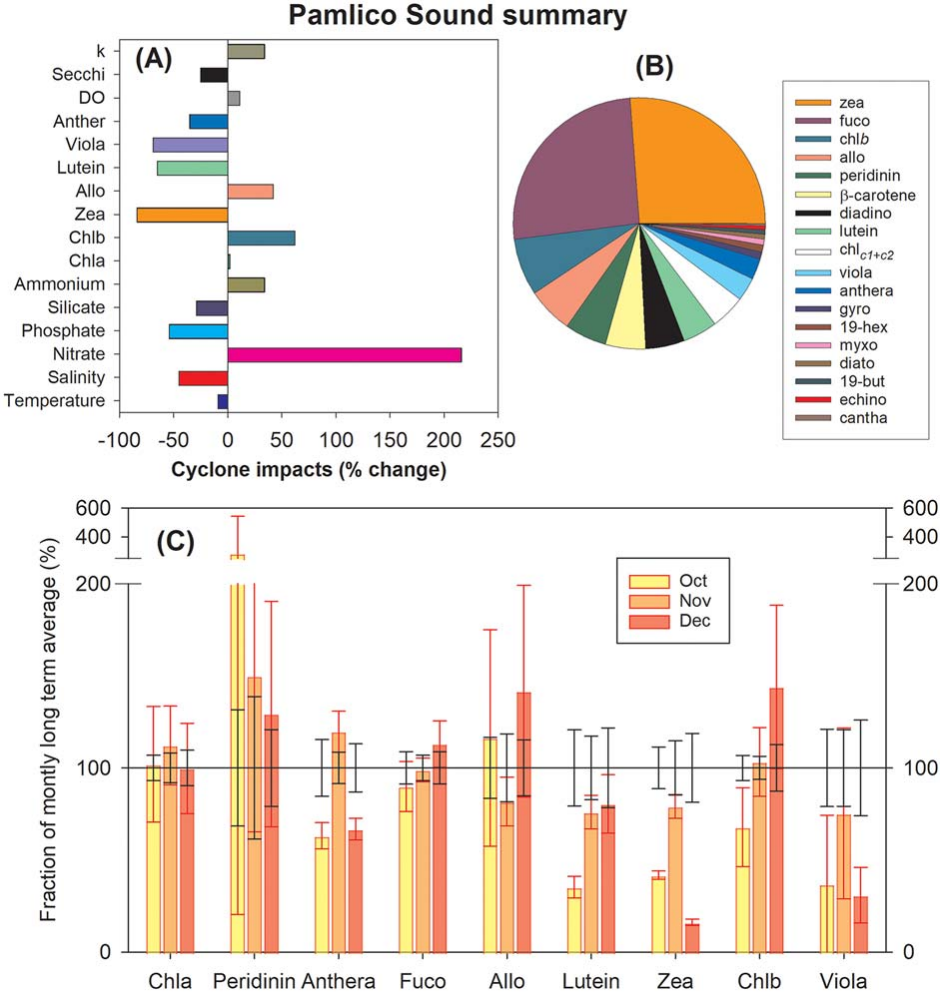
Pamlico sound summary. (**A**) The percent difference between the LTA monthly conditions (1994–2016) and conditions following the passage of Hurricane Matthew in 2016 for September (all except nitrate and ammonium) or November (nitrate and ammonium). (**B**) LTA relative accessory pigment concentrations for October (pie graph starts with zeaxanthin and goes counter clockwise to canthaxanthin). (**C**) Line at 100% is the LTA pigment concentrations for all stations and depths during October, November and December ±95% confidence intervals. Bars are the percentage of the LTA ± 95% confidence intervals observed in the same months during 2016.

Following the passage of Hurricane Matthew in October 2016, temperatures fell ~2°C or an average of 9% below the LTA and salinity was 45% below the LTA (Fig. 9A). The concentration of dissolved oxygen was also significantly impacted by the hurricane, rising to 11% above the LTA in October 2016 (Fig. 9A). A decline in water clarity accompanied the TC with Secchi disk depth falling 25% below the LTA to 0.97 m and the extinction coefficient rising 34% above the LTA in October 2016.

Phosphate concentrations were down 54% in October 2016 while silicate was down 29% from the LTA (Fig. 9A). Dissolved inorganic nitrogen concentrations, however, generally increased following Hurricane Matthew. There was a ~1 month delay for nitrate to rise 216% above normal and ammonium to reach 34% above normal (Fig. 9A).

The LTA pigment concentrations for Pamlico Sound indicate that diatoms, cyanobacteria, dinoflagellates, chlorophytes, prasinophytes and cryptophytes tend to dominate the October phytoplankton community (Fig. 9B). Across Pamlico Sound the overall biological response to Hurricane Matthew was apparently negligible, with the average concentration of chl*a* up 2% in October 2016 relative to the LTA (Fig. 9C). There were, however, both positive and negative changes that varied with taxa. The biomass of photosynthetic dinoflagellates (peridinin) reached 182% greater than normal in October 2016 and stayed above average through December. Cryptophytes (alloxanthin) tended to be above normal in October and December. Pigments that were below normal in October 2016 included lutein down 65%, chl*b* down 32% and violaxanthin down 69% suggesting chlorophytes were negatively impacted. Also significantly reduced for at least 3 months following the cyclone were cyanobacteria with zeaxanthin down 84% in December 2016.

#### Pamlico sound detailed results

Water temperatures were above normal from April to September 2016, but following the passage of Hurricane Matthew in October they fell ~2°C or 9% below the LTA (Supplementary Fig. S9A, Table II) although rising again above the LTA in November. Salinity persisted below the LTA throughout all of 2016 with the largest difference following the TC when salinity was down 45% to 9.9 (Supplementary Fig. S9B). The concentration of dissolved oxygen was generally below the LTA in mid-2016, but following the cyclone it rose to be 8.6 mg L^−1^ or 11% above the LTA in October 2016 (Supplementary Fig. S9C). A decline in water clarity also accompanied the cyclone with Secchi disk depth falling 25% below the LTA in October 2016 and remaining below 1.2 m for the remainder of the year (Supplementary Fig. S9E). The extinction coefficient (*K*_d_) rose 34% above the LTA in October 2016 although it declined to normal by December (Supplementary Fig. S9E).

Dissolved inorganic nitrogen concentrations generally increased following Hurricane Matthew (Supplementary Fig. S10). There was a ~1-month delay for nitrate to rise to 36 μg L^−1^ or 216% above normal (Supplementary Fig. S10A). Ammonium also increased in November and December 2016 to be >30% above normal (Supplementary Fig. S10B). In strong contrast, both phosphate and silicate were well below normal in October 2016 (Supplementary Fig. S10). Phosphate concentrations fell from a seasonal high of 47 μg L^−1^ to a low of 3.3 μg L^−1^ in November and were still below the LTA in December 2016. Silicate concentrations were also lower than normal, down 29% in October and falling steadily through the remainder of the year reaching an annual low of 22.8 μg L^−1^ in December (Supplementary Fig. S10D).

### Site 7. Port Aransas, Texas, USA

The site at Port Aransas is located on a tidal channel that receives water both from a ~160 km long estuarine system (Guadalupe, Mission-Aransas and Nueces estuaries) of south east Texas and the Gulf of Mexico (Anglès *et al.,* 2015). The estuaries are shallow (~1–3 m), often hypersaline (Buskey *et al.,* 1998) and predominantly separated from the ocean by low barrier islands. The Nueces, Aransas, San Antonio, Mission and Guadalupe Rivers flow into the estuary system, which is frequently nitrogen (N) limited (Pennock, 1999). In mid-June 2015 TS Bill made landfall just a few km north of Port Aransas, TX with sustained winds of 95 km h^−1^, producing a 0.9 m storm surge and 13 cm of rainfall at Port Aransas. Rainfall greater than 25 cm was reported from several regions in the catchment of the Gulf Intracoastal Waterway including Victoria County and Warton County where rainfall peaked at 33 cm. During May and June 2015, the Guadalupe River flow (USGS 08176500 Guadalupe River at Victoria, TX) was greater than normal, reaching 3.5 times the LTA (1935–2018) for June. Phytoplankton sampling occurred at the University of Texas Marine Science Institute pier, located in the ~7 km Port Aransas Channel at Port Aransas (27°50.296 N, 97°3.017 W). This channel connects the Mission-Aransas component of the Gulf Intracoastal Waterway with the Gulf of Mexico with tidal velocities from −1.5 to +1.8 m s^−1^. A 5 mL sample of near-surface water was collected every ~20 min, counted by an Imaging Flow Cytobot (Olson and Sosik, 2007) and compiled into monthly mean abundances of ~17 major taxa. The 17 taxonomic groups are a mix of species, genera, and one group of several taxa (DactFragCerataul = *Dactilyosolen fragilissimus* and other morphologically similar *Dactilyosolen* spp.), *Cerataulina* spp., and *Leptocylindrus danicus,*  Supplementary Table SI). Details of the location, methods and analysis of extreme events from this location can be found in Anglès *et al.,* 2015 and Fiorendino *et al.,* 2021 (this volume). The availability of high frequency samples made it possible to conduct a detailed statistical analysis that used each daily mean abundance as an independent replicate for a month. These data were used to compare the abundances of 17 selected taxa during the months of June, July and August 2015 with the LTA for the same months from the other 9 y in the data set (2008–2014, 2016, 2017).

#### Site 7. Port Aransas, Texas, USA. Summary results

**Fig. 10:**
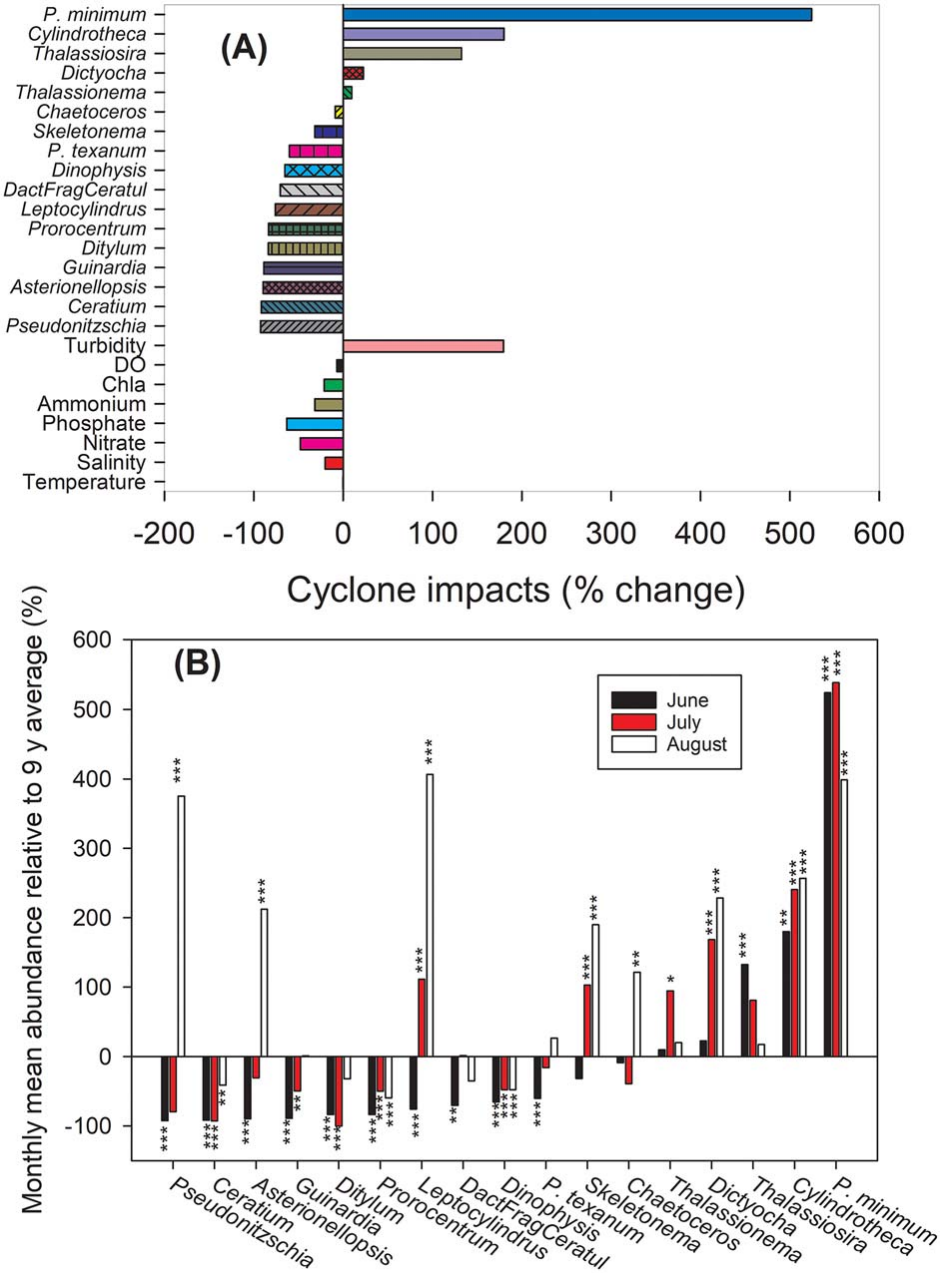
Port Aransas, Texas summary. (**A**) The percent difference between the LTA from (2008–2014, 2016, 2017 monthly conditions and those post the passage of TS Bill in June 2015. (**B**) The LTA relative abundance of dominant taxa for 1, 2, 3 months post the passage of TS Bill in 2015. Asterisks indicate a significant difference between June, July and August in 2015 and the LTA the same month (Mann Whitney rank sum test, ^***^P < 0.001, ^**^P < 0.01, ^*^P < 0.05). Details of taxa can be found in Supplementary Table SI.

At landfall TS Bill produced maximum sustained winds of 95 km h^−1^ and 299 mm rainfall in Jackson County, Texas. Following the passage of TS Bill in June 2015, the water temperature at Port Aransas fell only 0.1°C or ~0.4%, while salinity was down 20% (Fig. 10A, Table II). Nitrate, phosphate and ammonium concentrations were reduced 48%, 63% and 32%, respectively. Turbidity increased by 179%. Of the 17 most abundant taxa, 11 were reduced in abundances to be below their LTA by 9–92%. Five taxa increased in abundance rapidly in June 2015 relative to their LTAs; they were *Thalassionema, Dictyocha, Thalassiosira, Cyclindrotheca and Prorocentrum cordatum* (formerly, *P. minimum*) (Fig. 10A). *P. cordatum* was 524% above normal.

#### Site 7. Port Aransas, Texas, USA. Detailed results

All the 17 most abundant taxa were significantly different in abundance from their LTAs for at least one month between June, July and August 2015 (Fig. 10B). Only 5 of the 17 taxa, *Guinardia*, *Dactilyosolen fragilissimus* (plus other morphologically similar taxa), *P. texanum*, *Thalassionema* and *Thalassiosira,* returned to normal abundance after two months (Fig. 10B). Many of the diatoms, such as *Pseudonitzschia*, *Asterionellopsis, Leptocylindrus, Chaetoceros* and *Skeletonema*, were below their LTA abundance in June and recovered strongly to more than 100% above their LTA abundance by August. Three taxa, *Thalassiosira, Cyclindrotheca* and *P. cordatum* were all above their LTA abundance from June through August. *Cyclindrotheca* and *P. cordatum* were significantly (*P* < 0.01) above their LTA for all 3 months (Fig. 10B, Supplementary Table SI). *P. cordatum* was the species that appeared to thrive the most under these extreme conditions, with an average increase in abundance of 487% across June, July and August 2015.

### Site 8. The Pearl River Estuary, Hong Kong, China

The Pearl River Estuary (PRE) is located midway along the northern boundary of the South China Sea. The Pearl River is the world’s 13th largest river, discharging 3.36 × 10^11^ m^3^ largely in the monsoon season (Yin *et al.,* 2000; Zhang *et al.,* 2012). The buoyant brackish plume can extend up to 150 km alongshore (NW and SE) and reach the shelf break (Chen *et al.,* 2019). The extent of the low-salinity plume from the Pearl River depends on the season (Wong *et al.,* 2004), the wind, inshore sea level and the amount of runoff (Xue *et al.,* 2001). On a global scale, the western North Pacific and South China Sea regions are the most prone to severe cyclones with 31% of global TCs occurring in this region (Ramsay, 2018). Previous investigations of the impacts of cyclones showed initial declines in chl*a*, followed by a phytoplankton bloom and a drop in bottom water DO (Zhou *et al*., 2012). Typhoon Sam was the wettest TC to affect Hong Kong since records began in 1884, dropping over 616 mm of rain during 19–23 August 1999. Peak sustained winds of 96 km h^−1^ were recorded as the typhoon passed over the Territory. The heavy rain led to many instances of flooding and over 150 landslides throughout Hong Kong.

Data were obtained from the Hong Kong Environmental Protection Department (EPD) marine quality monitoring program for 10 stations in 3 regions sampled ~ monthly from 1986 to 2004 with 3 depths sampled at each station (Yin, 2002). The stations ranged in depth from 14 to 34 m (Supplementary Table SII), and were exposed to a gradient in the depth averaged salinity. The lowest average salinity was 29.2 at the 2 NM sites (near) in the PRE, 31.8 at the 4 SM (mid) sites in the coastal archipelago just east of the PRE and 32.5 at the 4 MM (far) sites further east from the Pearl River (Table II). A preliminary assessment of both the most commonly occurring taxa and the most abundant taxa at the level of species and genera revealed considerable inter-annual variability, and it was necessary to analyze the data at a higher level of taxonomic resolution to discern overall patterns of temporal change. Three taxonomic categories were used, diatoms, dinoflagellates and others. Several modeling studies have examined the impacts of cyclones on PRE plume dynamics, indicating that wave induced mixing is important for the distribution of the Pearl River runoff (Chen *et al.,* 2019; Zhang *et al.,* 2021).

#### Site 8. Hong Kong, China. Summary

**Fig. 11:**
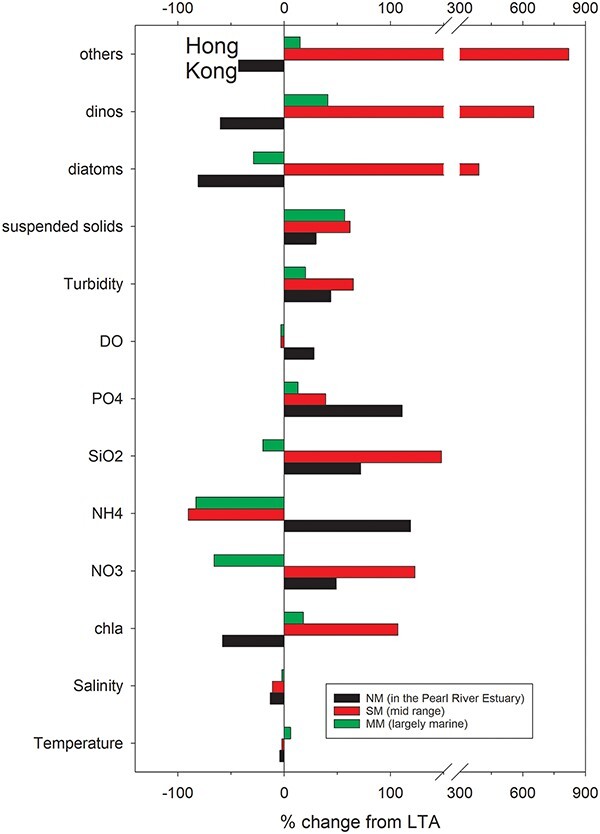
Summary of the Hong Kong waters. Data was obtained from the Hong Kong Environmental Protection Department (EPD) marine quality monitoring program consisting of 10 stations in 3 regions sampled ~ monthly with 3 depths sampled at each station. The 1986–2004 LTA monthly surface data were compared to the surface data obtained after the passage of Typhoon Sam in August 1999. The 2 NM sites (near) were in the PRE, the 4 SM sites (mid) in the nearby coastal archipelago and the 4 MM sites (far) furthest from the PRE with less input from the Pearl River.

As with other locations in this study, there were relatively modest changes in temperature associated with the cyclone. Following Typhoon Sam the temperatures deviated from their LTA in August by −4% to +6% or from −1.17 to +1.67°C (Fig. 11, Table II). Salinities varied somewhat more, with the LTA falling −13% at the stations nearest to the PRE (stations NM, Supplementary Fig. S11) and −2% in the more marine MM stations (Supplementary Fig. S17). Of the nutrients only phosphate was consistently greater than LTA in all three regions rising +110% at the more estuarine stations (Supplementary Fig. S11) to +13% at the more marine stations (Supplementary Fig. S17). Silicate and nitrate responded similarly; rising in the PRE (Supplementary Fig. S11) and more strongly in the middle (SM) region (Supplementary Fig. S14) from 49% to 148% but declining at the marine end (Supplementary Fig. S17). Ammonium fell strongly in both the middle and more marine regions. Both turbidity and suspended solids rose between 30% and 65% in all three regions (Supplementary Figs S12, S15, S18). Chl*a* and phytoplankton abundance fell 43% to 81% below their LTA in the PRE (Supplementary Figs S13 and S16, respectively) but exhibited a very substantial rise in both the middle (Supplementary Figs S15 and S16) and more marine (Supplementary Figs S18 and S19) regions with diatoms, dinoflagellates and other taxa increasing their abundances above the LTA by 391%, 653% and 820%, respectively (Fig. 11, Table II).

#### Site 8. Hong Kong, China. Detailed results

At the NM stations within the PRE the surface water temperatures had been greater than the LTA from February to June prior to Typhon Sam arriving in August (Supplementary Fig. S11A). In addition, the salinity was reduced from May to August while nitrate concentrations were elevated (Supplementary Fig. S11B and C, respectively). Phosphate concentrations were near normal in 1999 until after Typhon Sam passed in August when they jumped ~100% from 0.024 to 0.051 mg L^−1^ (Supplementary Fig. S11D) and a rise of similar magnitude was observed for silicate (Supplementary Fig. S11E).

In the PRE at the NM stations dissolved oxygen, turbidity, suspended solids and pH were all mildly elevated post Typhoon Sam but tended to be closer to normal by October (Supplementary Fig. S12). While chl*a* had been very close to the LTA in July it was only 50% of the LTA or 2.4 μg L^−1^ immediately following Typhoon Sam and declined even further to 0.75 μg L^−1^ in September before rising to be 3.5 μg L^−1,^ or 45% greater than the LTA in October (Supplementary Fig. S12A). The abundances of both diatoms and dinoflagellates were below normal in August and September 1999 rising to be close to normal in October (Supplementary Fig. S13).

In the island archipelago SE of the PRE at the SM (mid) stations the water temperatures fell marginally (−0.5°C) below the LTA in August 1999 while salinity dropped 3 (Supplementary Fig. S14). In August 1999 the nitrate, phosphate and silicate concentrations averaged ~100% greater than their LTA, at 0.34, 2.73, 0.02 mg L^−1^, respectively. Turbidity and suspended solids were up ~60% relative to the LTA in August 1999. The abundance of diatoms and dinoflagellates at the SM stations were much greater than the LTA in August 1999 with diatoms rising from ~9 × 10^6^ to 34 × 10^6^ cells L^−1^ (Supplementary Fig. S15).

In 1999 at the Hong Kong stations furthest from the PRE (MM) surface temperatures during August were 30°C, some 1.7°C greater than the LTA (Supplementary Fig. S17). Salinity, nitrate, phosphate and silicate concentrations were all within the 95% CI for the LTA during August 1999. Chl*a*, turbidity, suspended solids, pH were modestly elevated relative to their LTA and remained so for more than one month (Supplementary Fig. S18). The abundance of diatoms was slightly less than the LTA in August 1999 while dinoflagellate abundance was slightly greater (Supplementary Fig. S19), although neither were outside the 95% confidence intervals for the LTA.

### Overall results

**Fig. 12:**
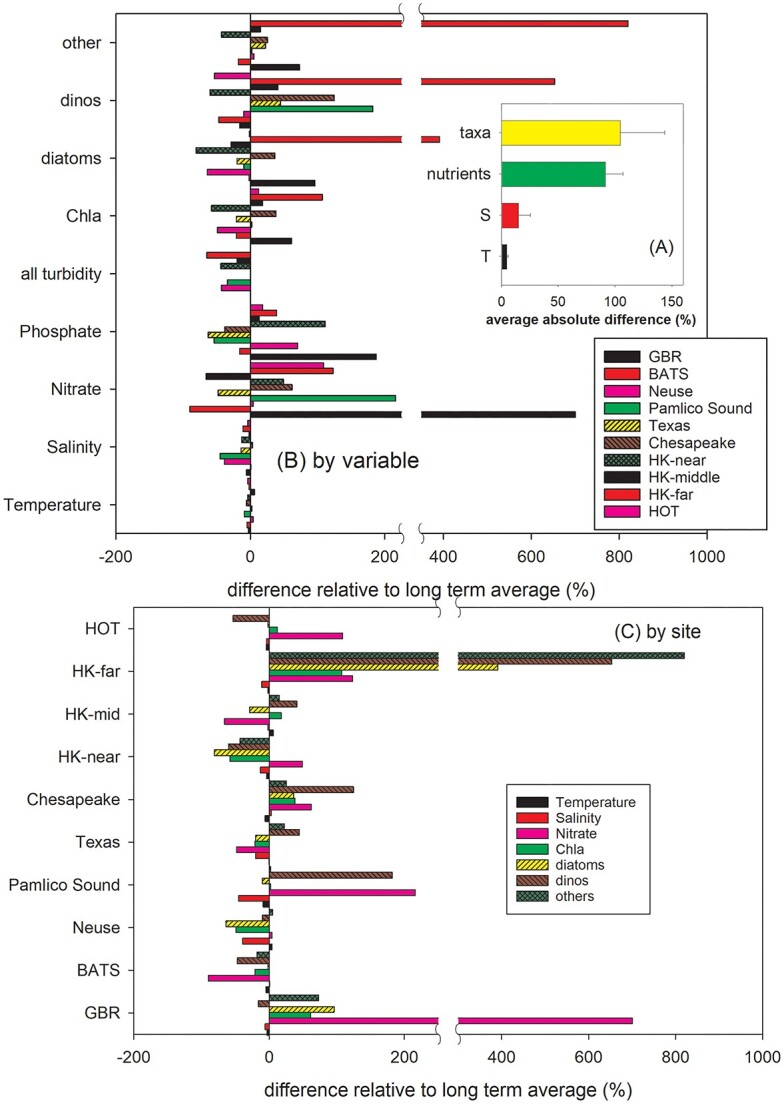
A summary of changes measured ~1 month after the passage of a TC at 10 different locations. (**A**) The absolute change expressed as a percentage of the LTA for temperature, salinity, combined nutrients and combined taxa. Error bars are 95% CI. (**B**) By variable, the % change in temperature, salinity, nitrate, phosphate, chl*a*, the abundances of diatoms, dinoflagellates and other taxa at 10 locations. (**C**) By location, the % change in temperature, salinity, nitrate, chl*a*, the abundances of diatoms, dinoflagellates and other taxa.

The post cyclonic differences relative to the LTA for temperature, salinity, nutrients, pigments, phytoplankton abundance, dissolved oxygen and water clarity ranged from −90 to +820% depending upon the region and the variable (Table II). Across all the sites and all reported variables, the only post TC changes that were sufficiently consistent in direction to be statistically significant were the 14.1% decrease in salinity and the 43% increase in light attenuation (Fig. 12B). Calculating the difference from the LTA as an absolute value and expressing it as a percentage of the LTA showed a relatively modest 4.6 ± 1.5% impact on temperature (Fig. 12A). Absolute differences in salinity post cyclone tended to be greater at 14.8 ± 11%. The largest differences were observed in nutrient concentrations and phytoplankton biomass (measure by the abundance of taxa or pigment concentration) where absolute differences averaged 90 ± 41% and 110 ± 40%, respectively (Fig. 12A).

At most sites post TC there was a mixture of positive and negative differences in water quality relative to their LTA (Fig. 12C). Some locations experienced predominately positive differences in nutrients and phytoplankton such as the Hong Kong sites just outside the PRE where nitrate concentrations, diatoms, dinoflagellates and other taxa all increased post TC. Although there were no consistent responses by all nutrients (nitrate, phosphate, silicate and ammonium) or by individual nutrients across all locations (Fig. 12B and C) the nitrate concentrations did rise at 7 of 10 locations including the Great Barrier Reef Lagoon, Pamlico Sound, along the mid channel of Chesapeake Bay, and some Hong Kong sites. The average post cyclone differences from LTA across all locations was +106% for nitrate +46% silicate +44% nitrite +28% phosphate and with an overall negative change of −16% for ammonium. Post TC the greatest difference from the LTA for nitrate + nitrite, phosphate and ammonium was at the surface at a single site the GBR lagoon where they averaged 297% greater. The NRE had the smallest average difference (+12%), and at −62% BATS nutrients remained well below their LTA (Fig. 2C) post TC.

## DISCUSSION

In this study, the impacts on environmental conditions brought about by TCs included a small decrease (averaged ~4% or 0.88°C) in temperature which we judge to have relatively little direct impact on phytoplankton ecology (Thompson *et al.,* 1992; Thomas *et al.,* 2016). Similarly, the observed average decline in salinity of 13% would mean that salinity would largely remain within the range tolerated by most estuarine and coastal species (Brand, 1984; Balzano *et al.,* 2011). In late summer when most TCs tend to occur the seasonal insolation is still relatively high. However, at 41%, the average reduction water clarity following a TC is likely to have a negative impact on some taxa. A reduction in irradiance could favor those species that can better regulate their depth such as dinoflagellates and cryptophytes or those species that grow well at lower irradiances. Late summer is also a period when many aquatic ecosystems are relatively nutrient deplete (Malone *et al*., 1988; Rudek *et al.,* 1991) suggesting that of the reported variables, the generally large increase in nutrient concentrations following a TC is likely to have the most significant impacts on phytoplankton ecology. As was observed for Sagami Bay (Tsuchiya *et al.,* 2014) following a TC both nutrients and phytoplankton accumulated in Pamlico Sound, the Great Barrier Reef lagoon, seaward of the PRE (Zheng and Tang, 2007) and at the more seaward end of Chesapeake Bay. Post TC these receiving water bodies had nitrate concentrations averaging 275% greater than their LTAs. The phosphate and silicate differences from their LTA were more variable than nitrate post TC, but still averaged +35% and +111%, respectively. TCs clearly have potential to reduce seasonal nutrient limitation (Rudek *et al.,* 1991) and support new phytoplankton blooms (Ye *et al.,* 2013).

Oceanic phytoplankton at mid latitudes generally tend to be strongly nutrient limited often for much of the year (Arteaga *et al.,* 2014) and commonly form a deep layer of maximum abundance where growth may simultaneously be constrained by light (Cullen, 2015; Cornec *et al.,* 2021). Following the passage of TCs through the open ocean, remote sensing has detected surface cooling (Bates *et al.,* 1998) and an increase in chl*a* that has been positively correlated with wind speed and negatively correlated with mixed layer depth (Pan *et al.,* 2018). Cooling and mixing result in a deepening of the surface mixed layer and injection of nutrients from depth (Babin *et al.,* 2004, Song *et al*., 2007). There is also growing evidence that TCs interacting with eddies of a similar direction of rotation strongly induces upward transport of nutrients into the euphotic zone (Lü *et al.,* 2020).

In the open ocean off Bermuda (BATS), the seasonal decline of stratification and temperature in the euphotic zone was following the passage of Hurricane Nicole was more rapid than the normal seasonal progression. In addition, the nutrient concentrations at depth had been less than normal before the TC, but they increased faster than normal throughout autumn and into early winter. In contrast, within days of TS Guillermo passing near HOT there was >100% increase in silicate, phosphate and nitrate concentrations between 100 and 150 m. These nutrient concentrations slowly declined over 4 months to near normal by December. At least at BATS, and possibly at HOT, it is likely the TCs delivered another potentially limiting nutrient, iron (Holzer *et al.,* 2016; Sedwick *et al.,* 2020). Significant iron arrives at BATS during summer as dust carried by storms from Africa and enhanced vertical mixing is the primary mechanism for delivery to the phytoplankton at depth.

In the open ocean, the post TC responses by phytoplankton tended to be slow and observed primarily at depth. Both HOT and BATs sites had more chl*a* than normal at ≥100 m for several months post event. As reported for previous TC events at BATS (Sweeney *et al.,* 2003) Hurricane Nicole also stimulated a strong increase in zeaxanthin concentration suggesting the primary phytoplankton response was by cyanobacteria. At HOT for several months post storm event (August to October 2015) there were increases in *Prochlorococcus* and various pico-eukaryotes at ~125 m. Overall, where nutrient concentrations within the euphotic zone of the oligotrophic ocean rise, but still remain low relative to the average uptake potential of phytoplankton, then it is the smallest photoautotrophic cells such as *Synechococcus, Prochlorococcu*s and picoeukaryotes that should benefit (Raven, 1998). Following TC Nicole’s passage at BATS, the vertical particulate fluxes of fresh phytodetritus, zooplankton and microbial biomass increased by 30–300% at 1500 m and 30–800% at 3200 m (Pàmies *et al*., 2019), suggesting a significant phytoplankton bloom over a large area and a strong ecosystem response to this TC.

In the short-term, advection appeared to be the dominant factor determining changes in phytoplankton abundance at the more riverine end of the estuaries studied. High amounts of rainfall in the catchment followed by discharge of floodwaters into the estuary made the upper Chesapeake Bay waters fresher, nutrients were increased and chl*a* initially declined 59%. Similarly, phytoplankton initially declined within the PRE and in the NRE. In the Pearl River Estuary and upper Chesapeake Bay, the taxa that recovered in abundance within one or two months were predominantly euryhaline pennate and centric diatoms including *Skeletonema* spp. Given the general decline in irradiance the rapid increase in abundance of these taxa may also reflect their capacity to grow well in turbulent and low light conditions (Margelef, 1974, Mortain-Bertrand *et al.,* 1988). The NRE revealed an unusual longitudinal distribution of photosynthetic dinoflagellates post TC with a peak in peridinin ~50 km from the freshwater end of the NRE. These data suggest that some dinoflagellates may have a greater capacity to resist the relatively rapid rise in advection associated with a TC possibly using their motility to seek out intermediate salinities (Cohen, 1985; Hall *et al.,* 2015) and may not be as strongly advected.

The receiving waters of the Great Barrier Reef Lagoon, Pamlico Sound, Hong Kong sites further from the Pearl River Estuary, lower Chesapeake Bay and the combined Guadalupe, Mission-Aransas and Nueces estuaries experienced increased abundances of various common diatoms in the months following their storm event potentially reflecting their rapid growth rates following a nutrient pulse, under euryhaline conditions and relatively low irradiance (Mortain-Bertrand *et al.,* 1988; Litchman and Klausmeier, 2008). In the Great Barrier Reef Lagoon pennate diatoms increased 422%. In Chesapeake Bay the early positive responders were *Skeletonema* spp. > *Dactyliosolen fragilissimus*. In the coastal waters off Hong Kong diatom abundances increased 390% and included the genera *Thalassiosira* > *Chaetoceros* > *Pseudonitzschia* > *Skeletonema*. In the Guadalupe, Mission-Aransas and Nueces estuaries the greatest increases among the diatoms observed at Port Aransas were by *Cylindrotheca* > *Thalassiosira* > *Thalassionema*. The Port Aransas site provided the best temporal resolution in this study and it clearly showed a strong positive response in the abundance of the following genera: *Asterionellopsis, Chaetoceros, Cylindrotheca, Leptocylindrus, Pseudonitzschia and Skeletonema,* over 1–3 months.

Increased abundances of dinoflagellates were also reported following a TC for Sagami Bay (Tsuchiya *et al.,* 2014) and for other TC events impacting the Neuse-Pamlico Sound (Hall *et al.,* 2013). In the receiving waters of Pamlico Sound, Hong Kong (mid), Chesapeake Bay and the Port Aransas site dinoflagellate abundance showed strong positive responses following the TC events reported herein; rising by an average of 251%. The rapid rise in dinoflagellate abundances detected in these water bodies may largely reflect advection and translocation. Indeed, at these four locations dinoflagellate abundance quickly rose and then declined post TC to approximately normal over periods of weeks to months. The abundance of *Katonatum rotundatum* and *Gymnodinium* spp. increased by 112% and 31% respectively in Chesapeake Bay immediately following the TC. Dinoflagellates also increased their abundance rapidly to ~6× times greater than the LTA in middle stations from Hong Kong. Several dinoflagellate taxa including *P. cordatum* showed large positive increases in abundance at Port Aransas. *P. cordatum* was possibly advected seaward from the combined Guadalupe, Mission-Aransas and Nueces estuaries or, potentially, arriving from more oceanic waters during tidal influx. The longer-term increase (~3 months) in dinoflagellates observed at Port Aransas could reflect dinoflagellate growth on inorganic and organic nutrients from the upstream estuaries, as well as potential tidal inputs of nutrients and cells from the downwelling conditions offshore (Fiorendino *et al.,* 2021, this volume).

## CONCLUSIONS

The most significant impacts of a TC were changes to nutrient concentrations and phytoplankton abundances. In the open ocean phytoplankton responses to TCs tended to be deep, with the greatest increase in nutrients and phytoplankton between 100 and 200 m and lasting up to 3 months. In the open ocean the increase in pigment concentrations, cyanobacteria and picoeukaryotes suggest the environmental impacts of a TC were not sufficient to eliminate the advantages of small cells (Raven *et al.,* 2005). It seems likely these impacts extend along cyclone tracks potentially making them widespread and globally significant.

In contrast, at the riverine end of coastal estuaries such as the upper Chesapeake Bay, the NRE and those Hong Kong stations in the Pearl River Estuary; the predominate response was a rapid and strong decline in salinity and phytoplankton indicating the TC impacts on phytoplankton were initially dominated by advection. At these sites, the environmental conditions and phytoplankton community generally returned to near normal within 2–3 months. At the more intermediate coastal water bodies where the residence times were longer, such as the Great Barrier Reef Lagoon, the lower Chesapeake Bay, Pamlico Sound, the Guadalupe, Mission-Aransas and Nueces estuaries and the Hong Kong stations further from the Pearl River Estuary, there were high loads of sediment and nutrients plus phytoplankton arriving from upstream. Over the next 1–3 month these receiving waters usually developed phytoplankton blooms most often dinoflagellate, diatom and sometimes cryptophyte blooms, which elevated chl*a* concentrations significantly for up to 3 months.

## Supplementary Material

FigS1_fbac062

FigS2_fbac062

FigS3_fbac062

FigS4_fbac062

FigS5_fbac062

FigS6_fbac062

FigS7_fbac062

FigS8_fbac062

FigS9_fbac062

FigS10_fbac062

FigS11_fbac062

FigS12_fbac062

FigS13_fbac062

FigS14_fbac062

FigS15_fbac062

FigS16_fbac062

FigS17_fbac062

FigS18_fbac062

FigS19_fbac062
