# Supplementary figures and images for: Tropical cyclones: what are their impacts on phytoplankton ecology?
Source: J Plankton Res. 2022 Nov 21;45(1):180–204. doi: 10.1093/plankt/fbac062 (PMC9897026; doi:10.1093/plankt/fbac062)

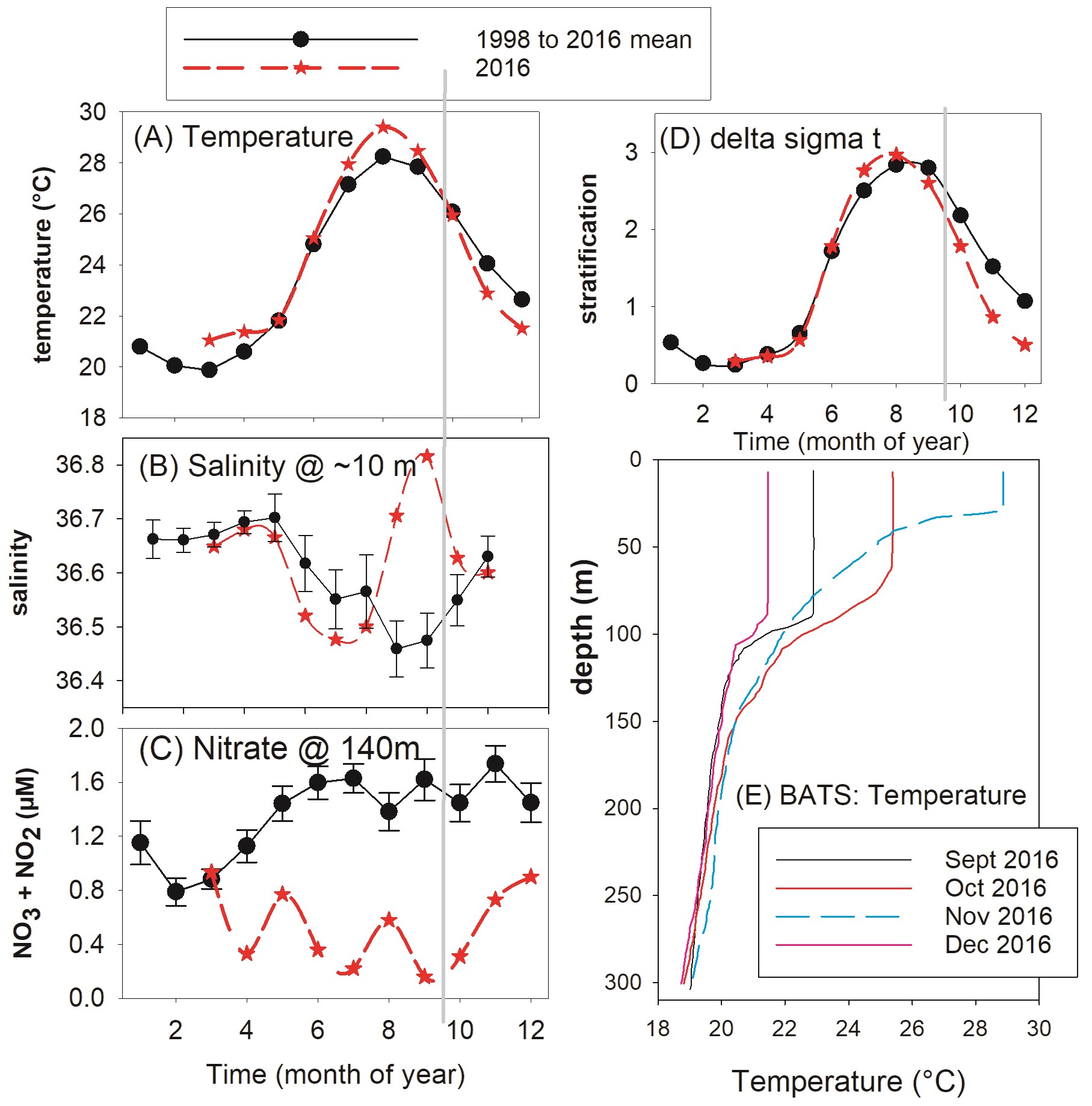

Supplement: FigS1_fbac062 [file figs1_fbac062.jpeg]

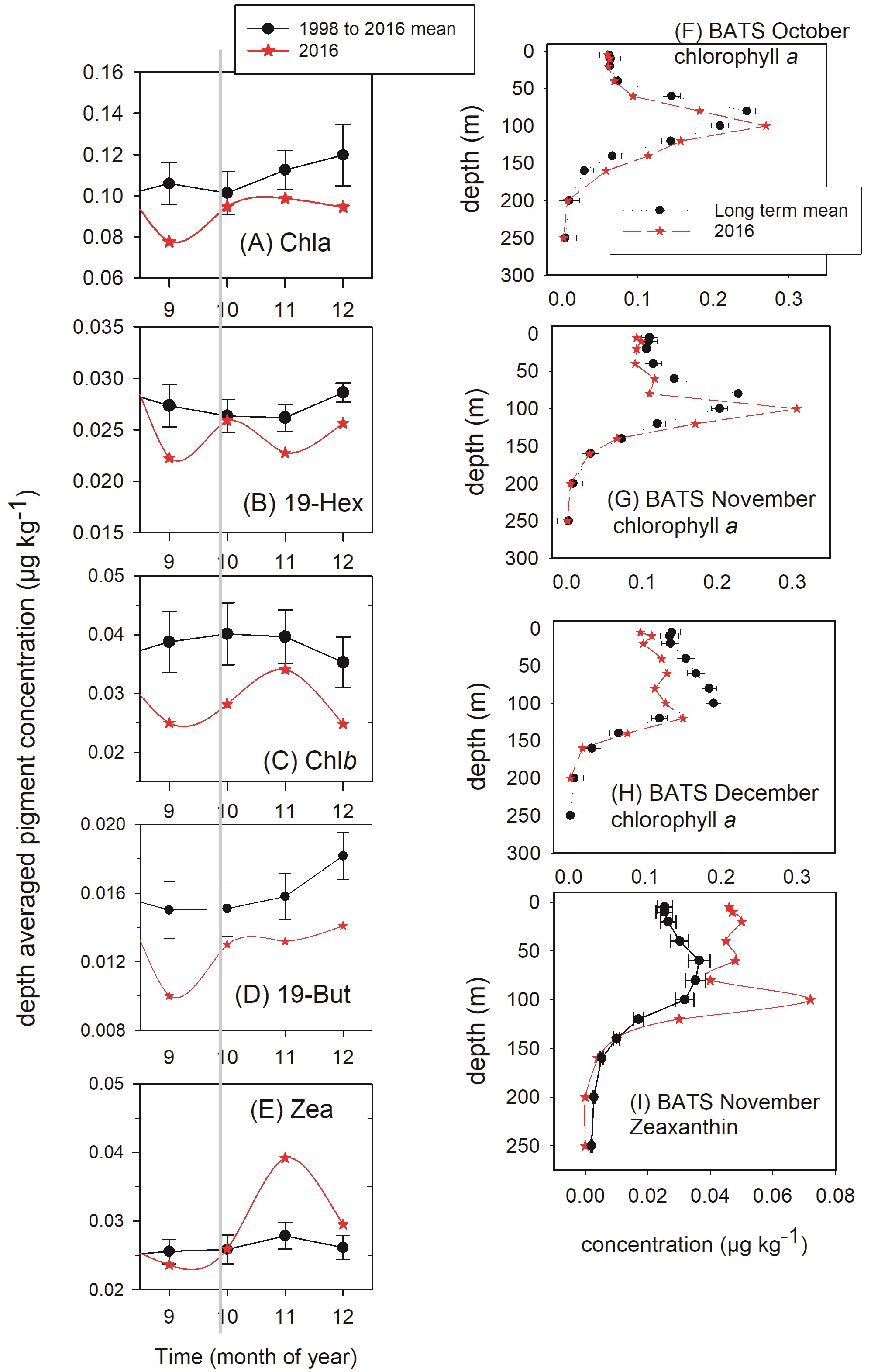

Supplement: FigS2_fbac062 [file figs2_fbac062.jpeg]

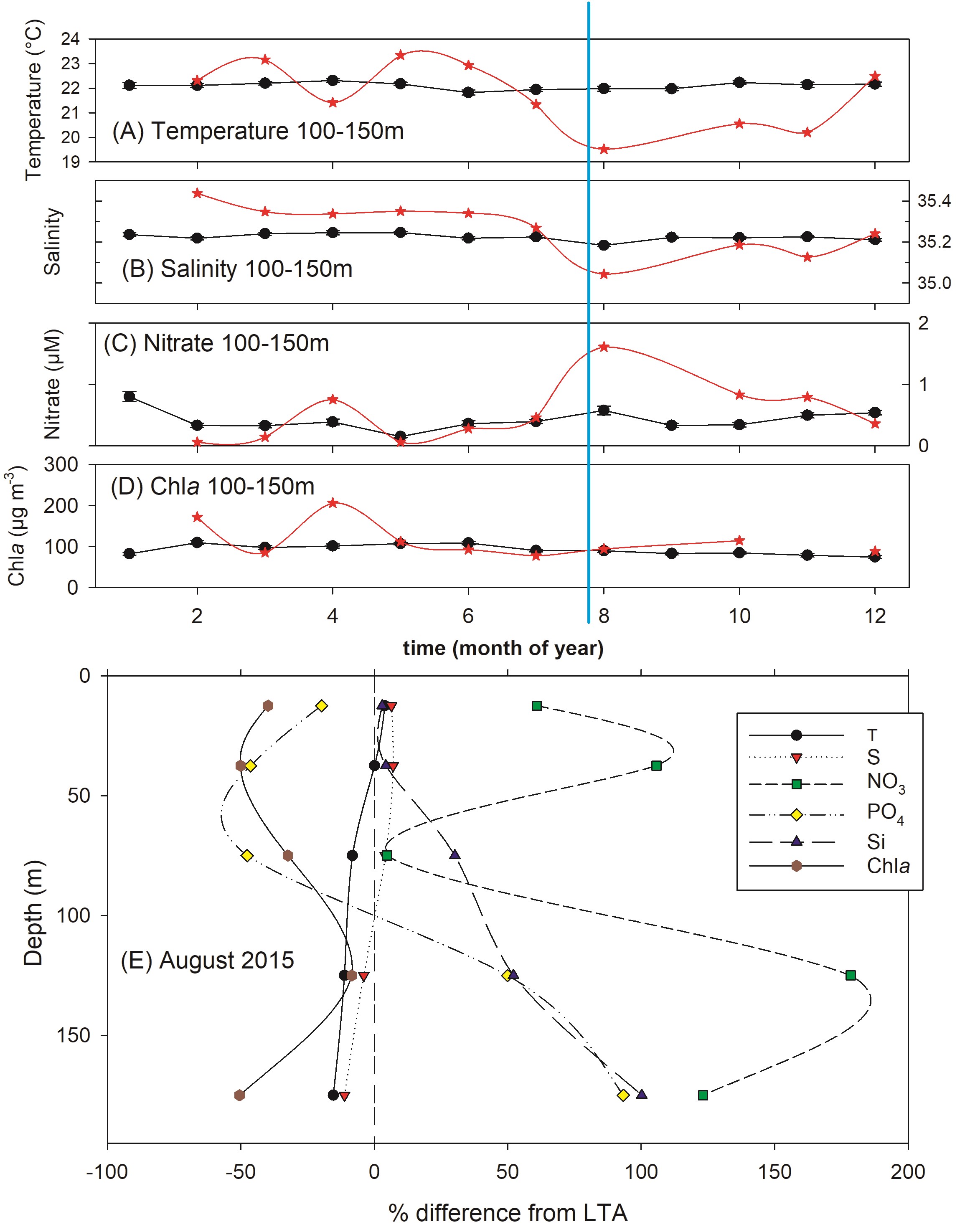

Supplement: FigS3_fbac062 [file figs3_fbac062.jpeg]

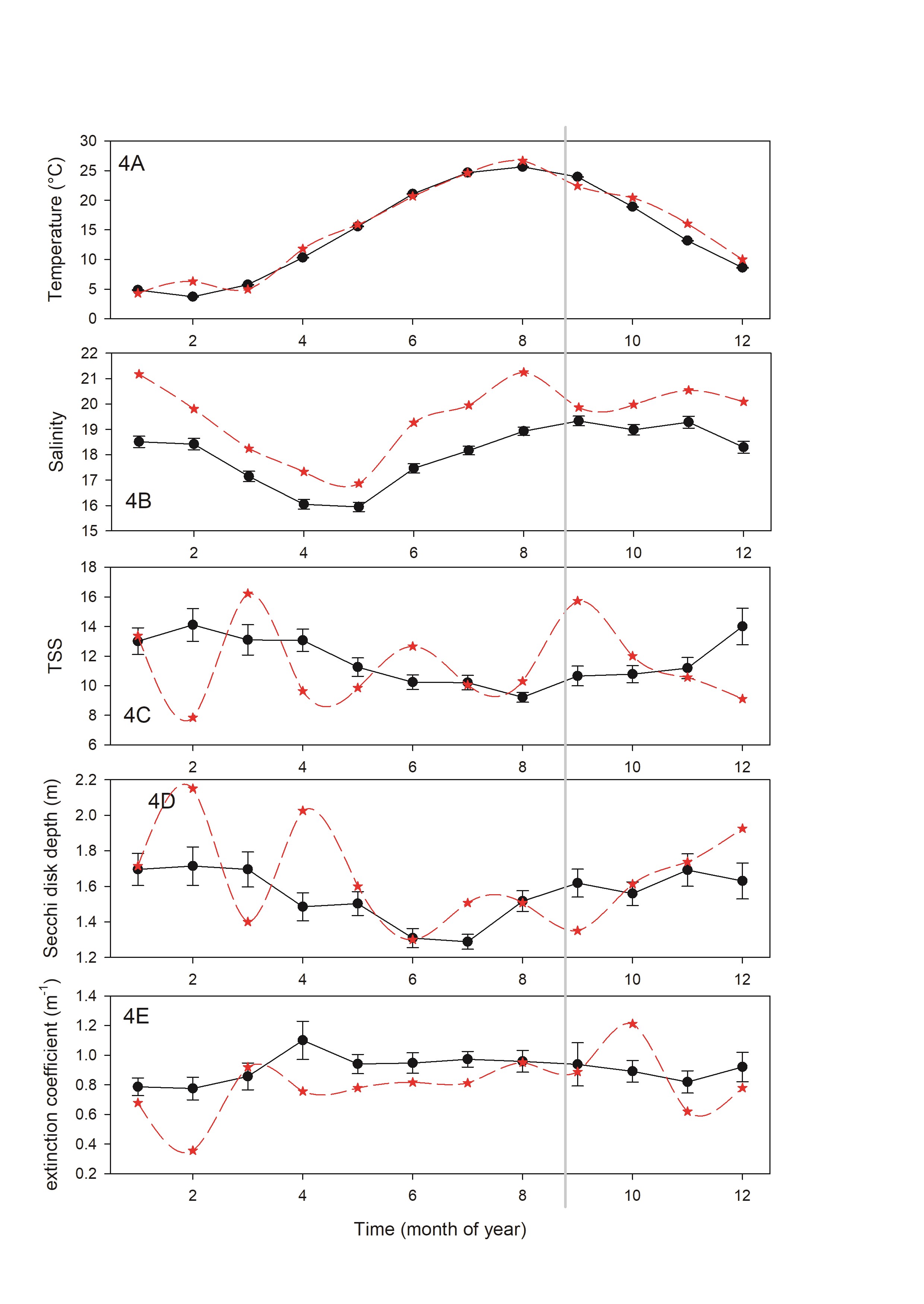

Supplement: FigS4_fbac062 [file figs4_fbac062.jpeg]

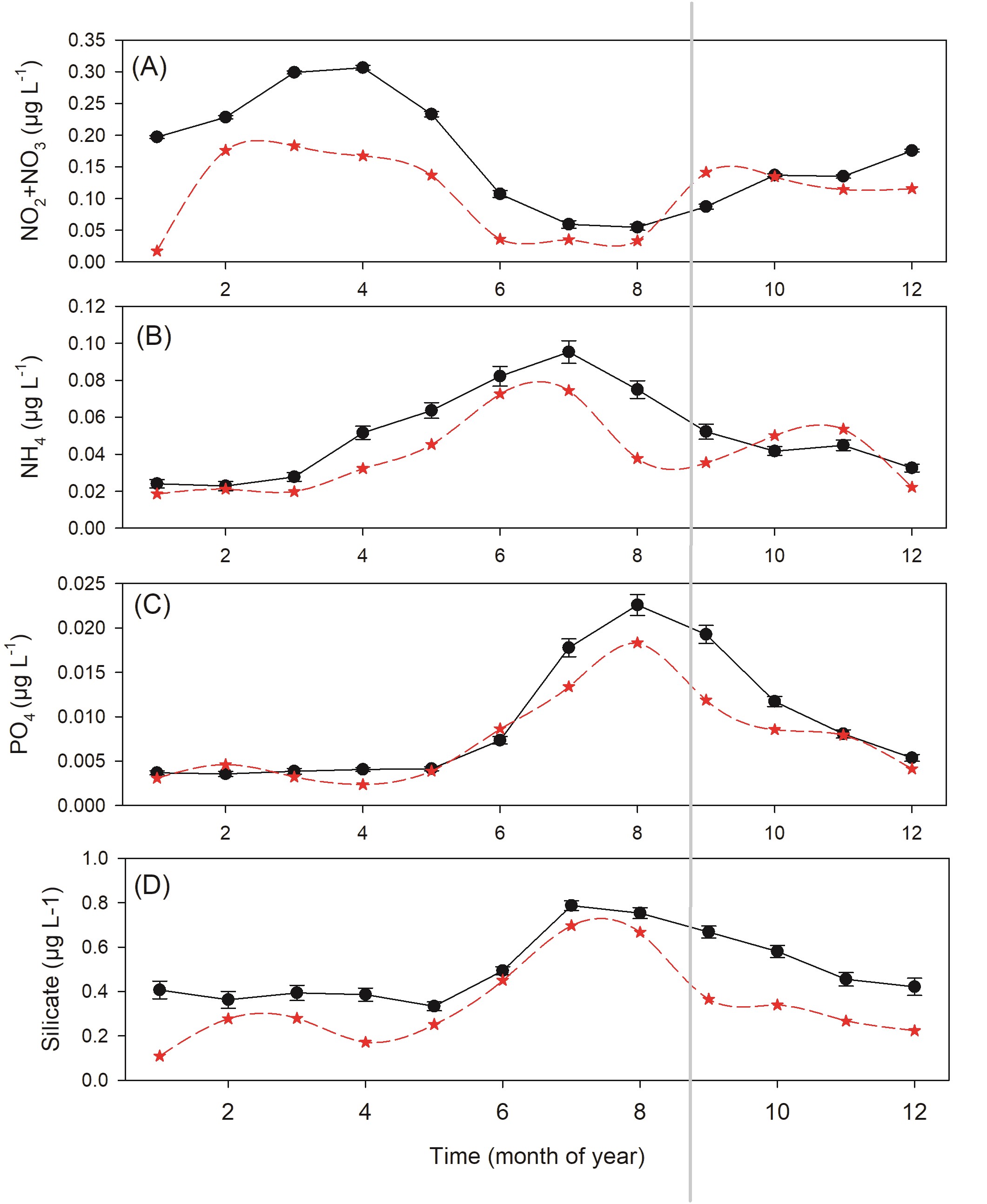

Supplement: FigS5_fbac062 [file figs5_fbac062.jpeg]

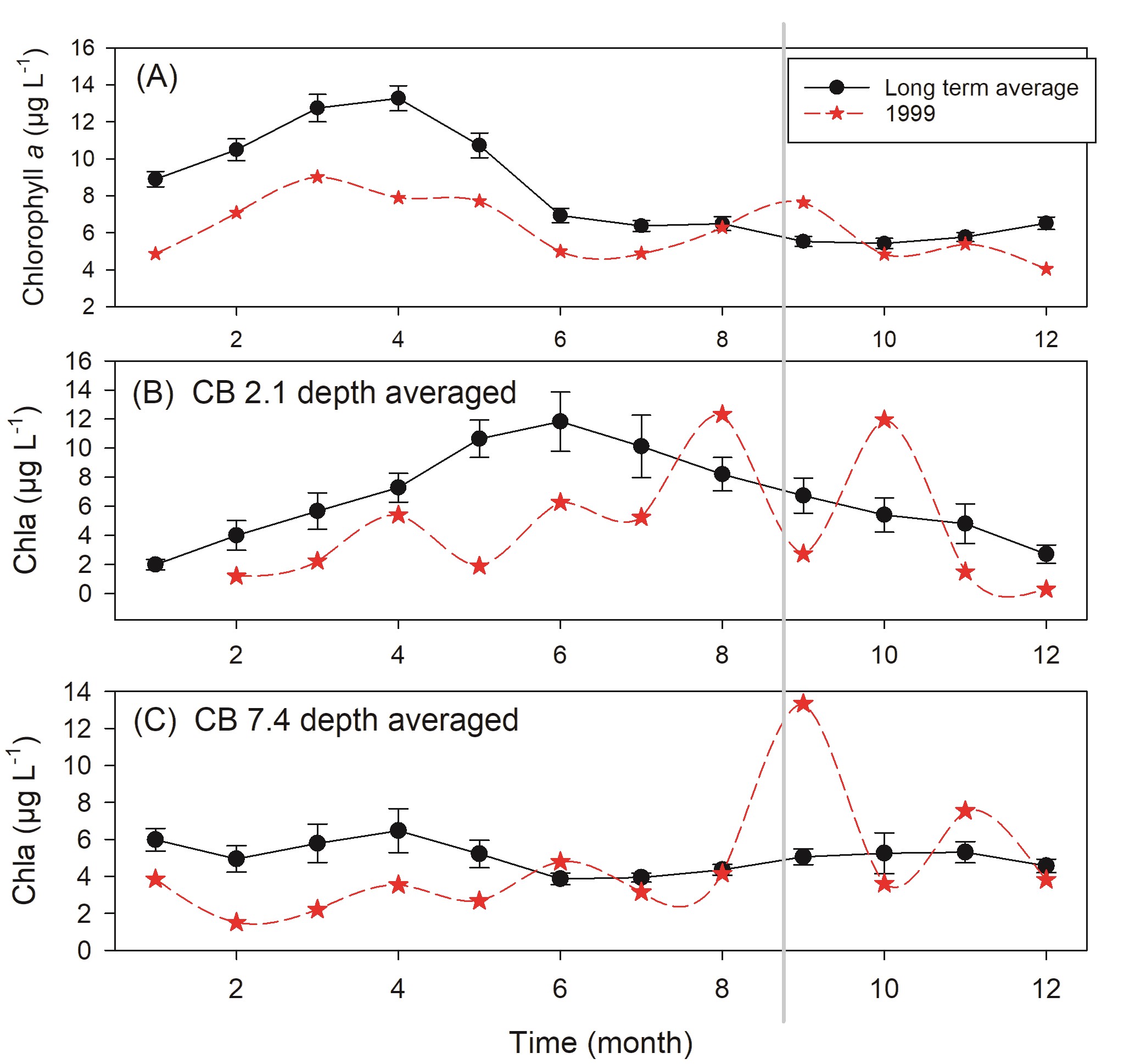

Supplement: FigS6_fbac062 [file figs6_fbac062.jpeg]

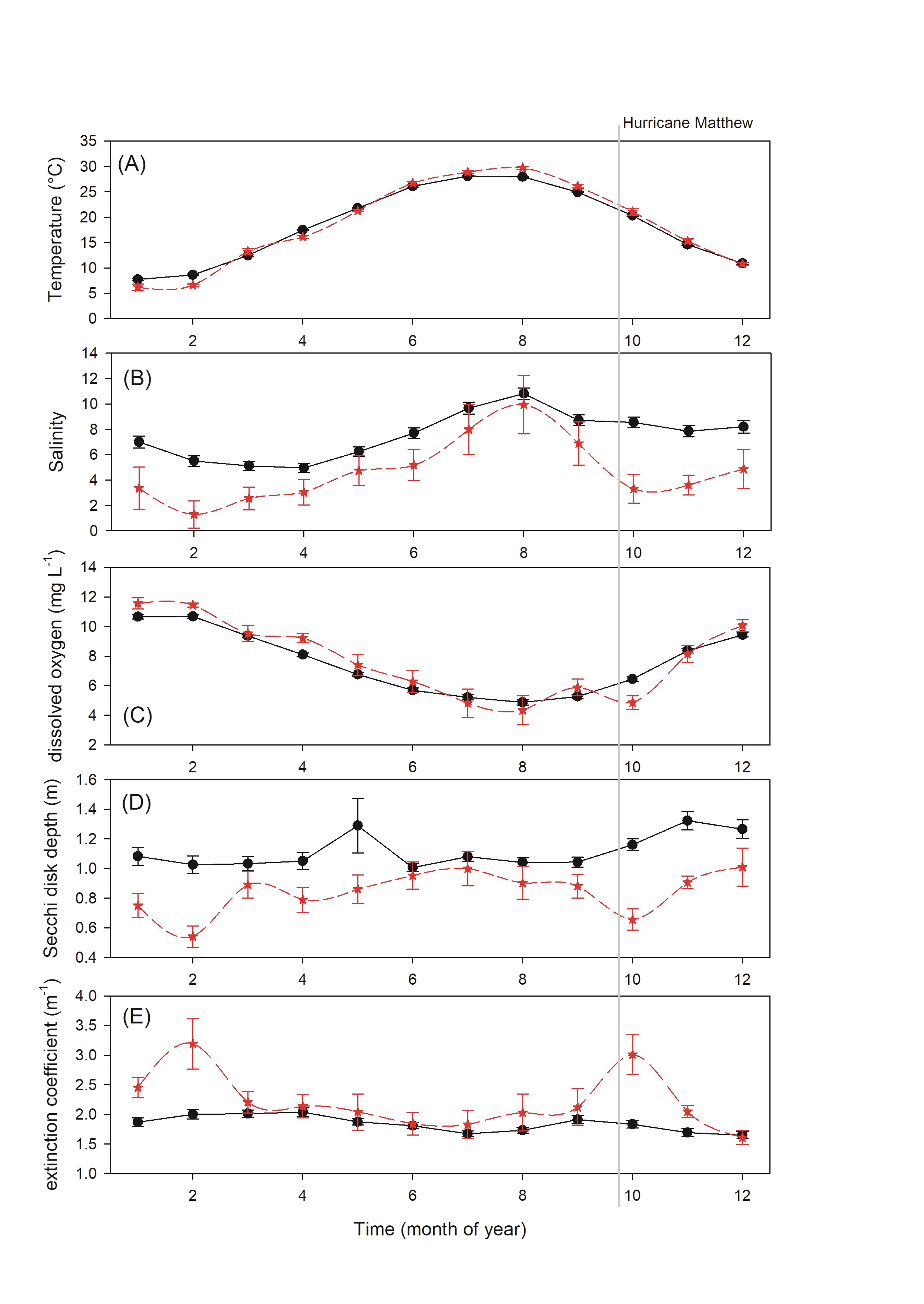

Supplement: FigS7_fbac062 [file figs7_fbac062.jpeg]

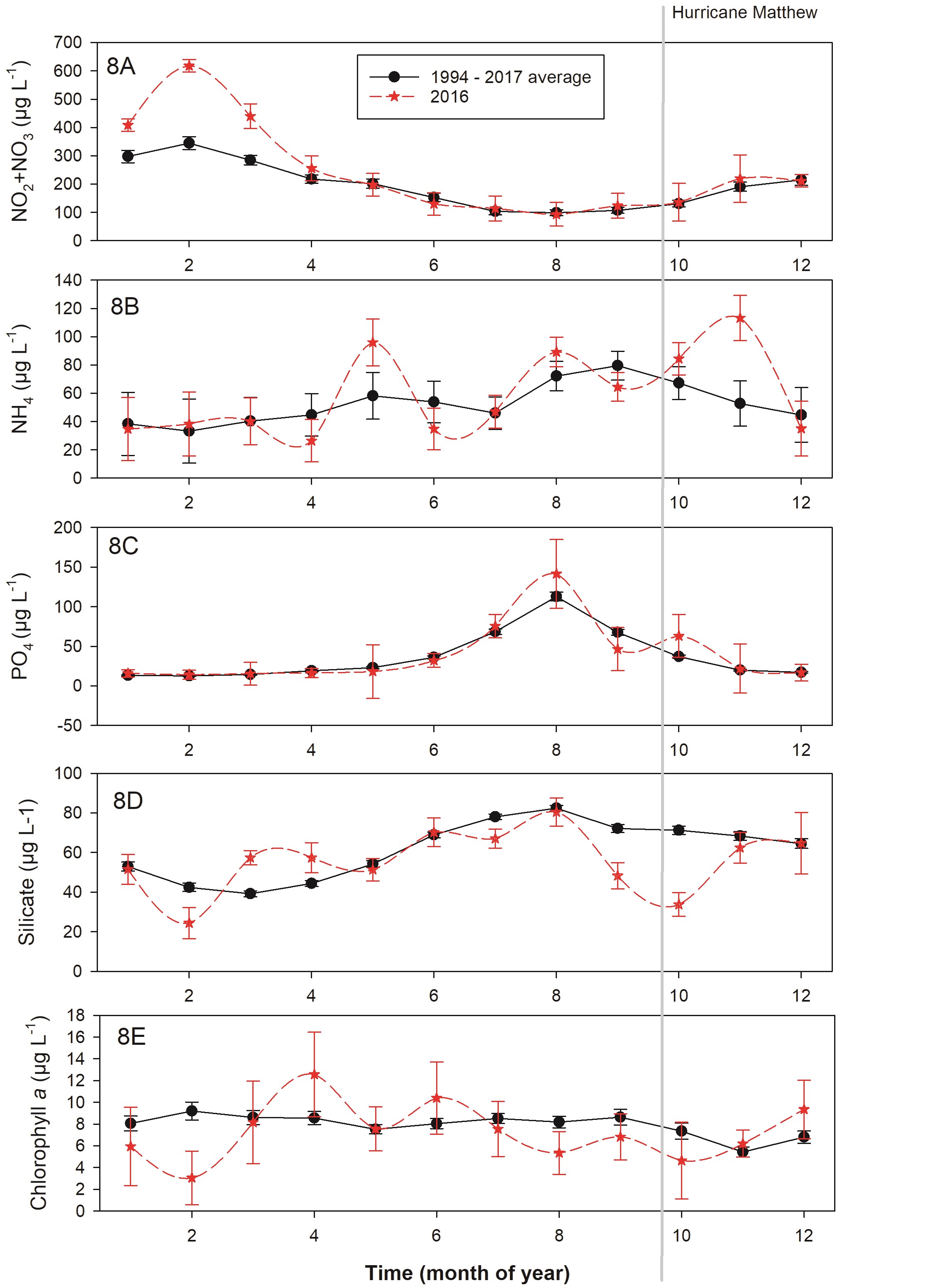

Supplement: FigS8_fbac062 [file figs8_fbac062.jpeg]

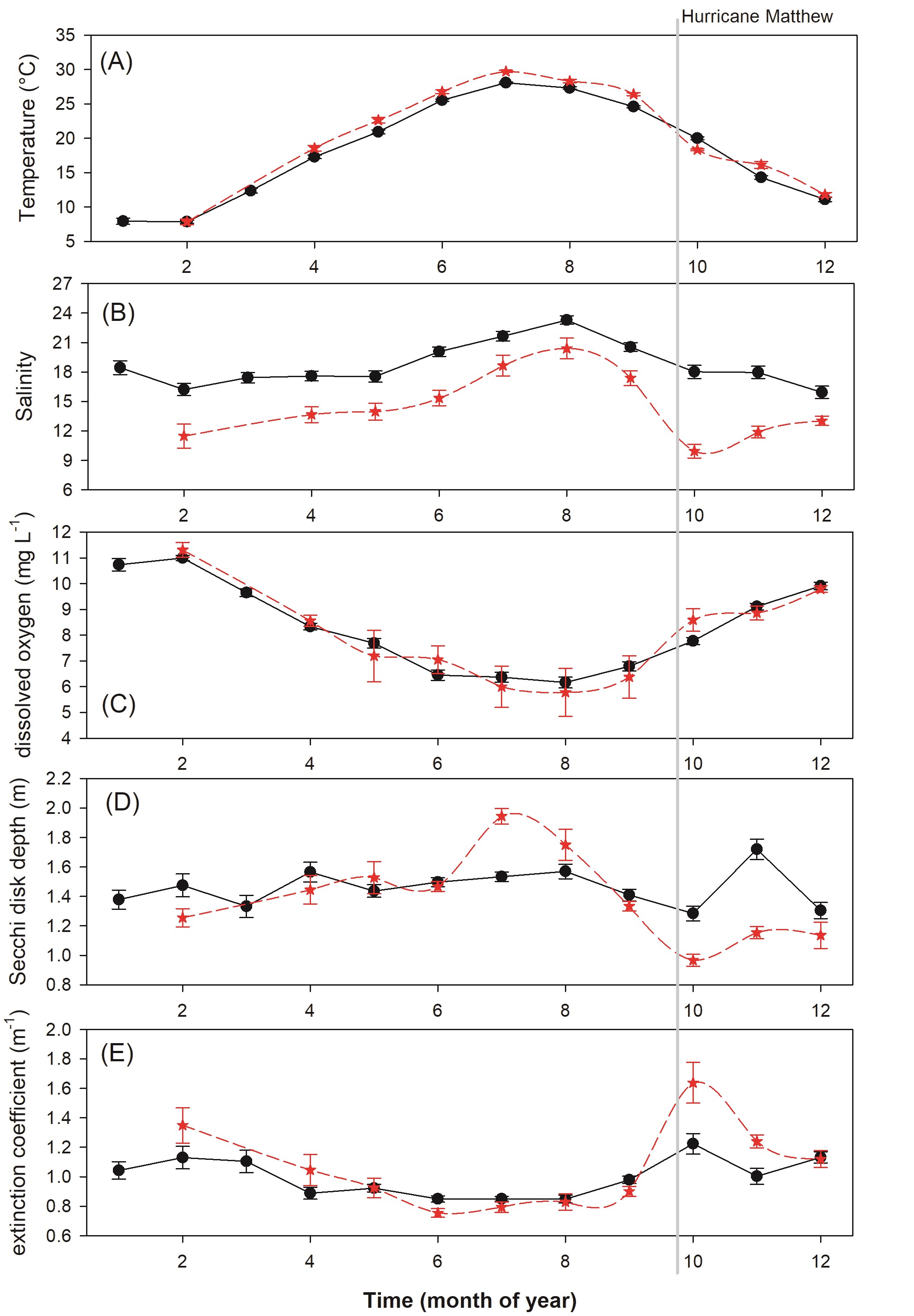

Supplement: FigS9_fbac062 [file figs9_fbac062.jpeg]

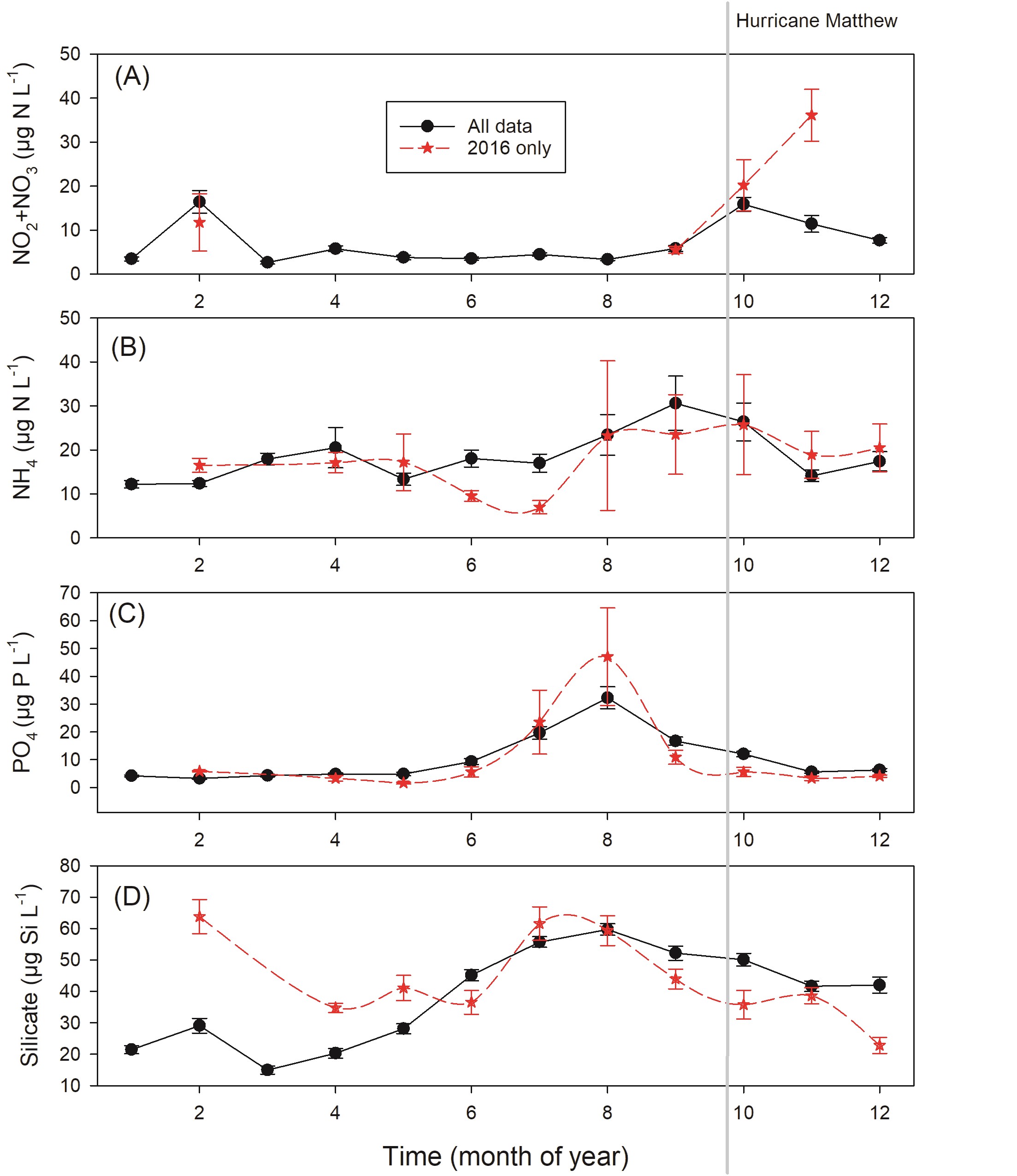

Supplement: FigS10_fbac062 [file figs10_fbac062.jpeg]

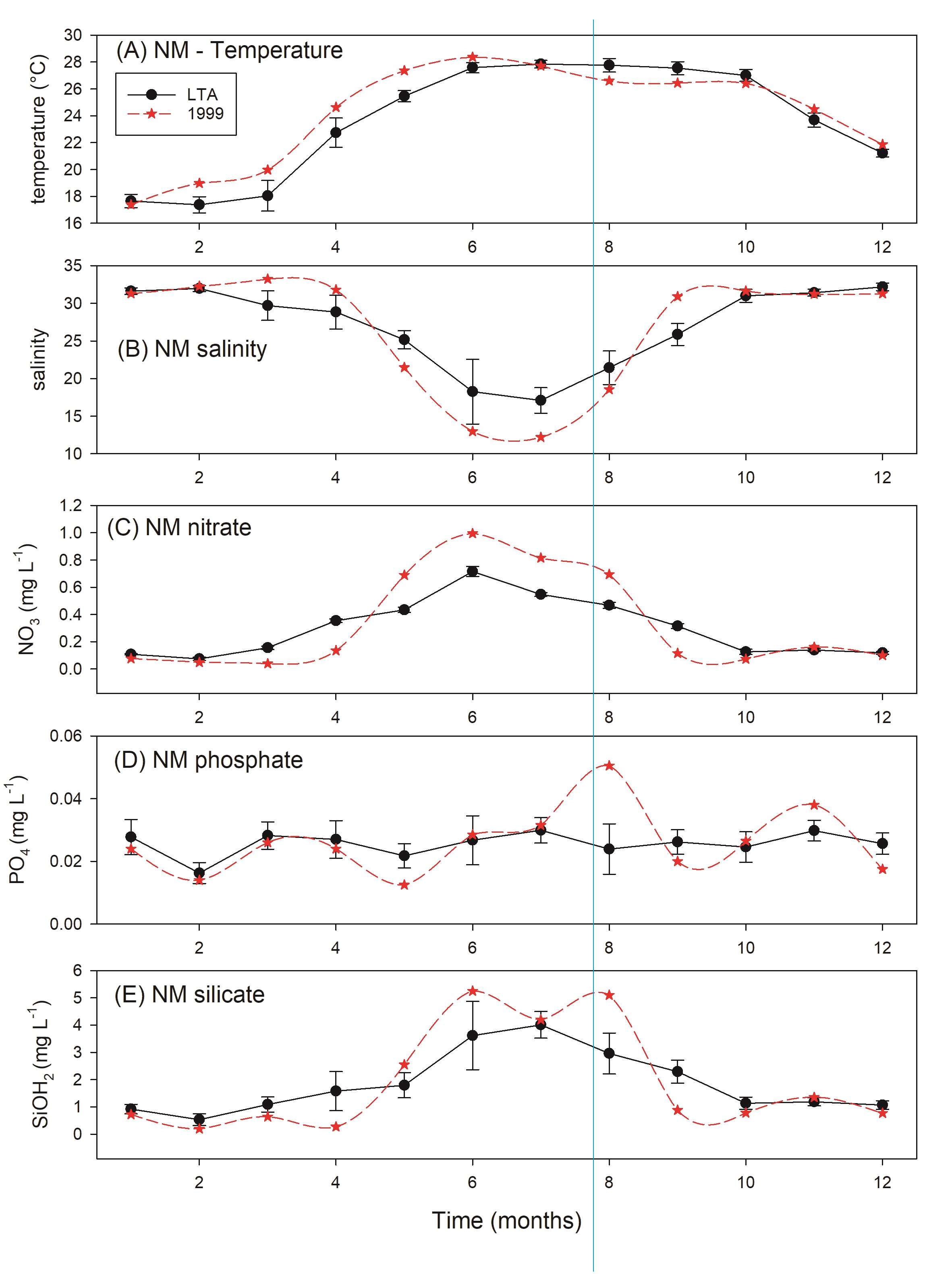

Supplement: FigS11_fbac062 [file figs11_fbac062.jpeg]

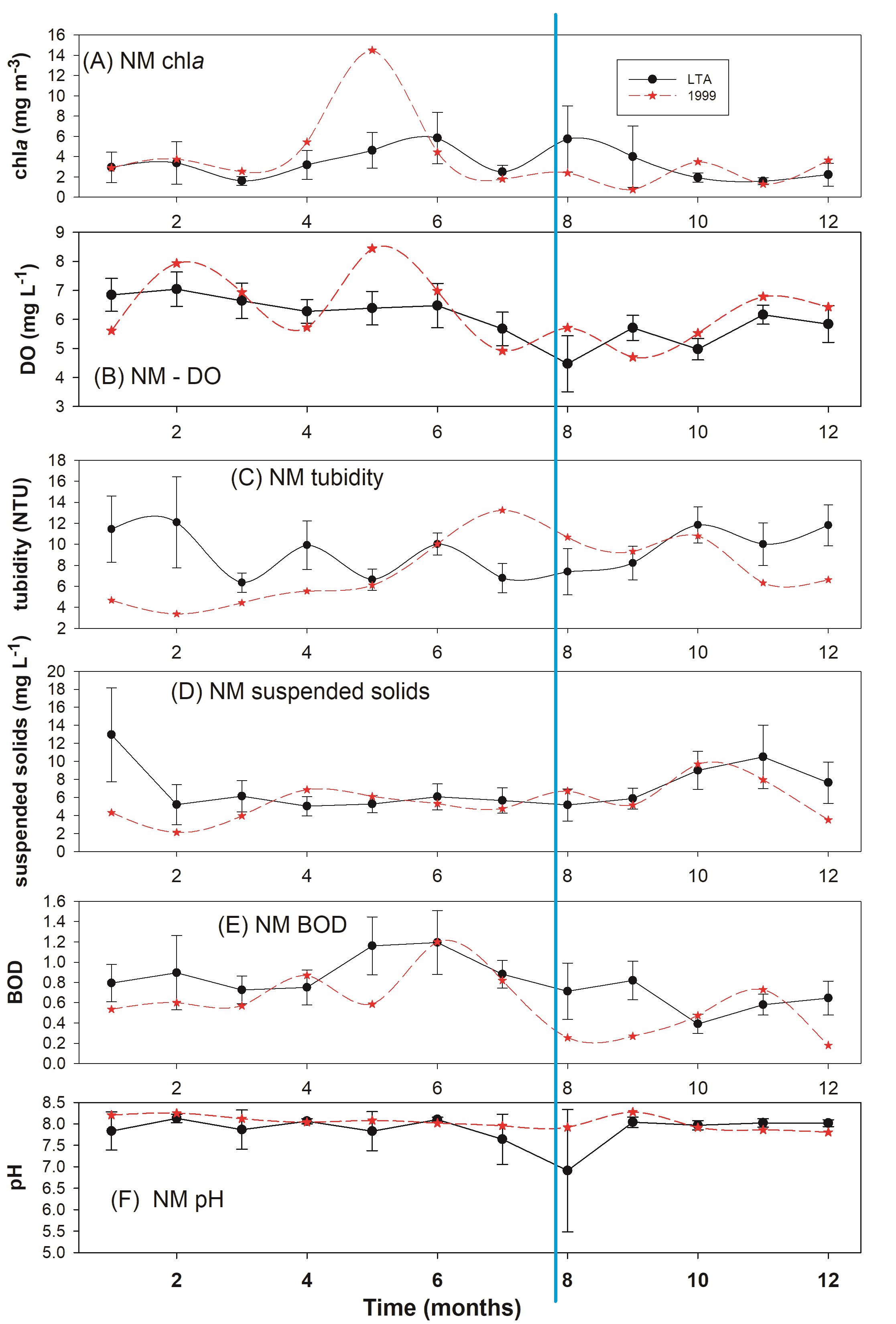

Supplement: FigS12_fbac062 [file figs12_fbac062.jpeg]

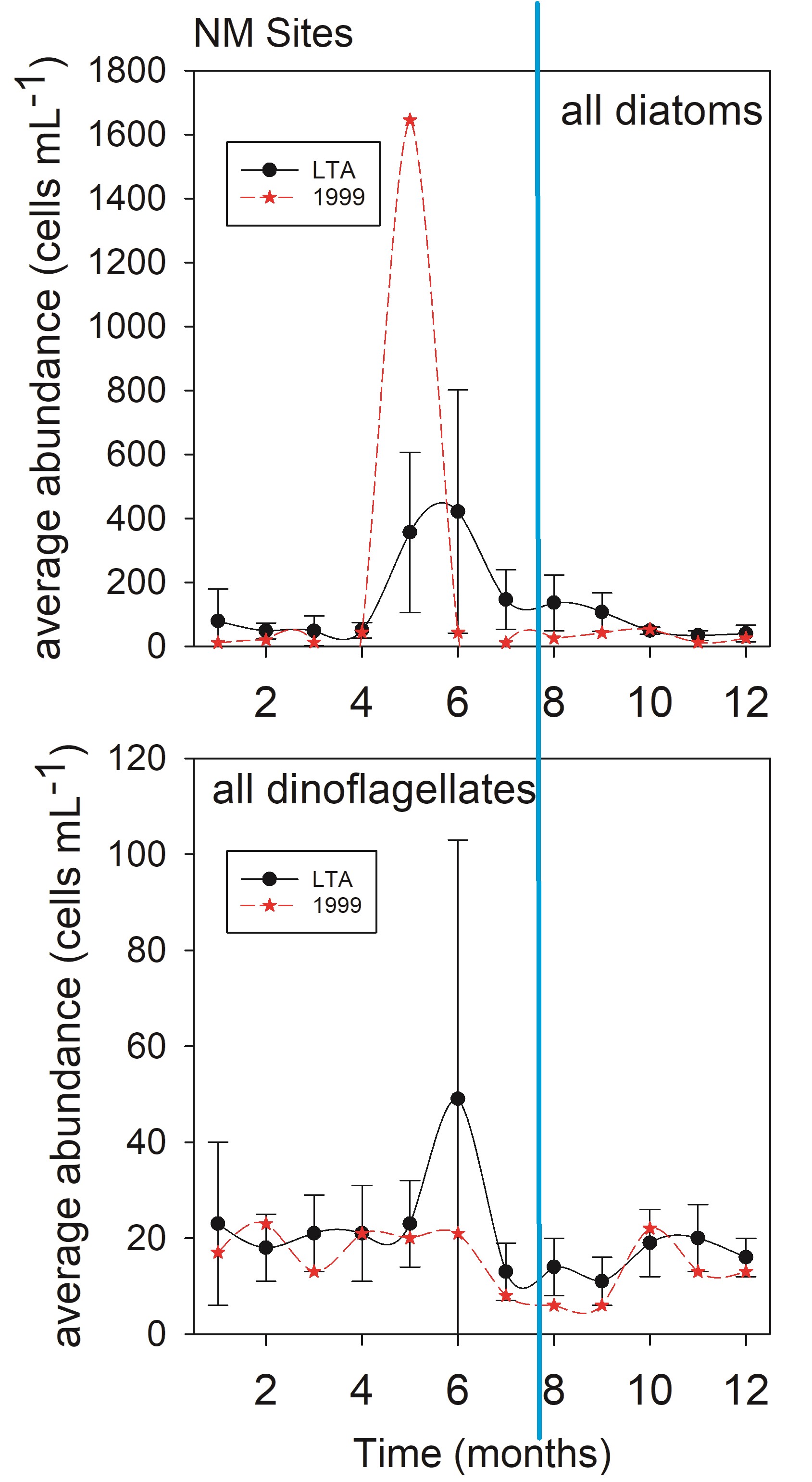

Supplement: FigS13_fbac062 [file figs13_fbac062.jpeg]

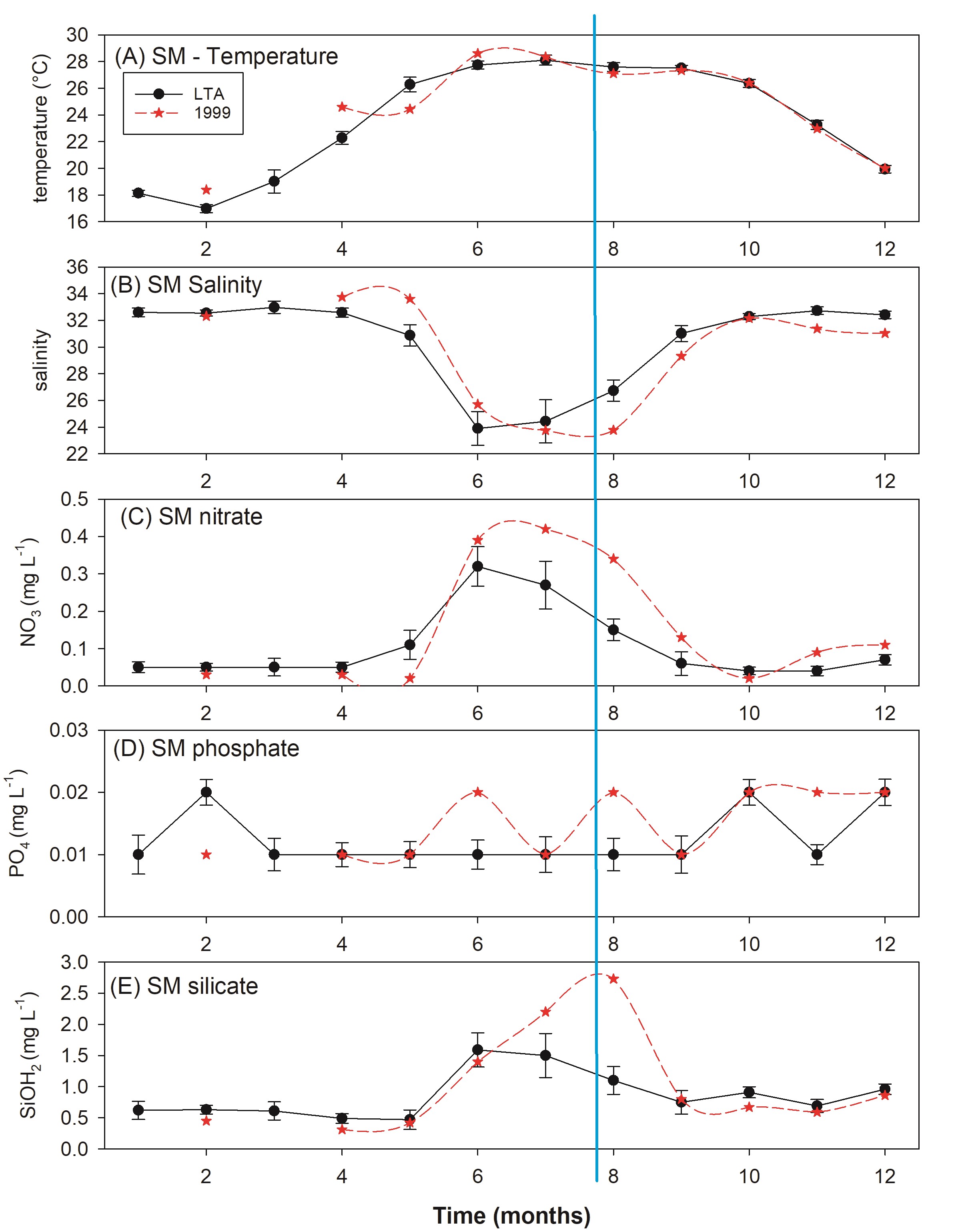

Supplement: FigS14_fbac062 [file figs14_fbac062.jpeg]

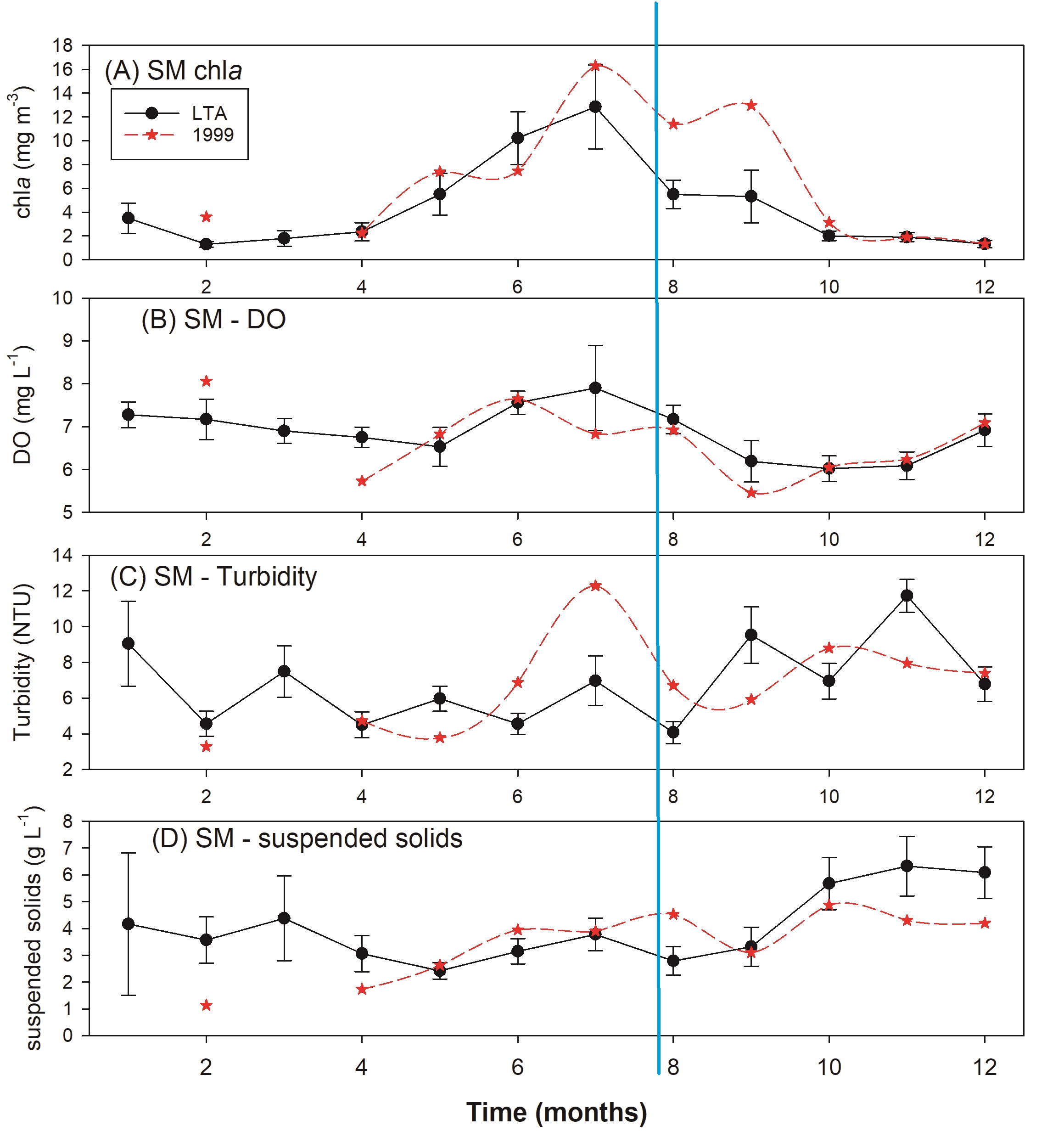

Supplement: FigS15_fbac062 [file figs15_fbac062.jpeg]

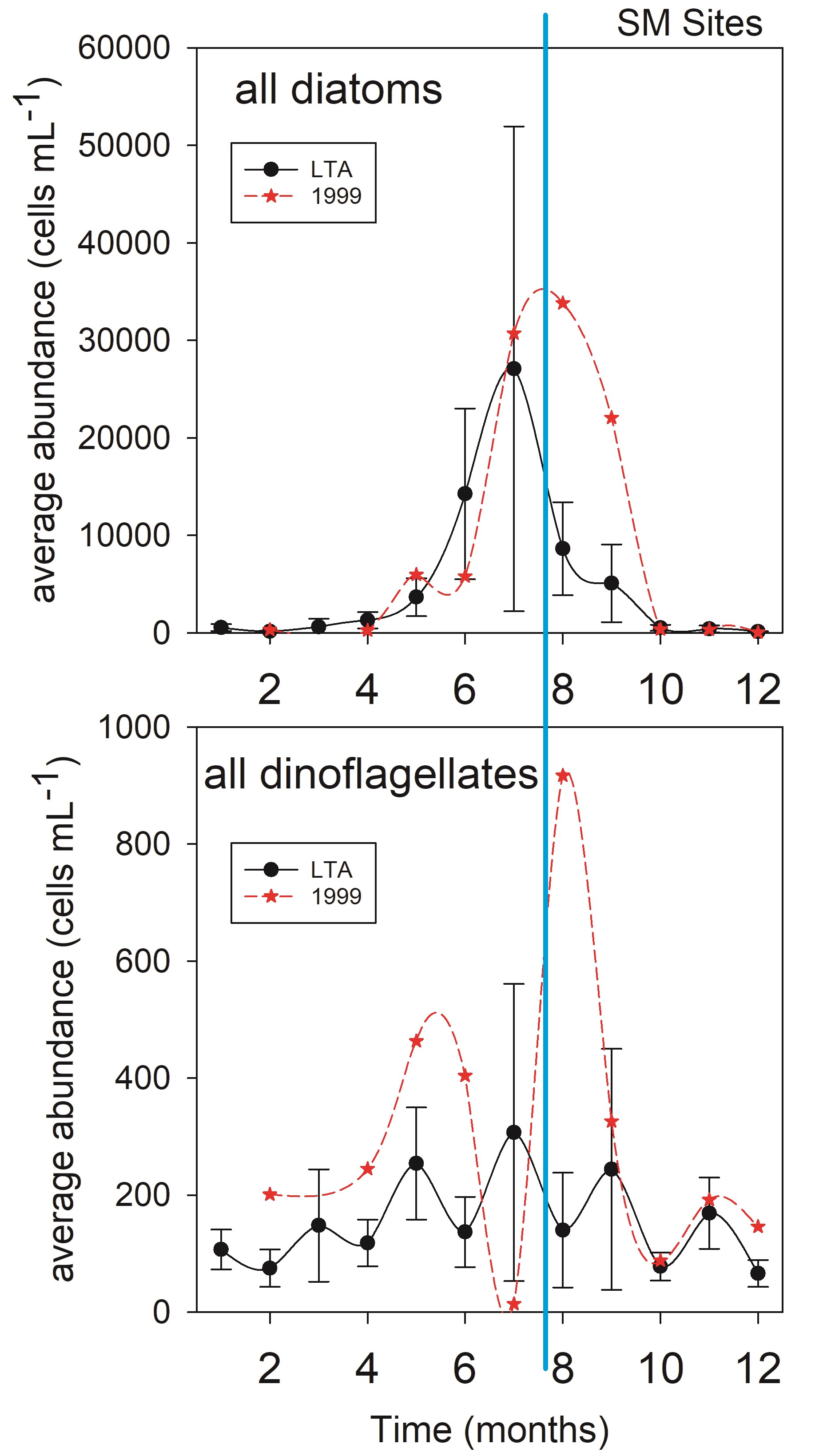

Supplement: FigS16_fbac062 [file figs16_fbac062.jpeg]

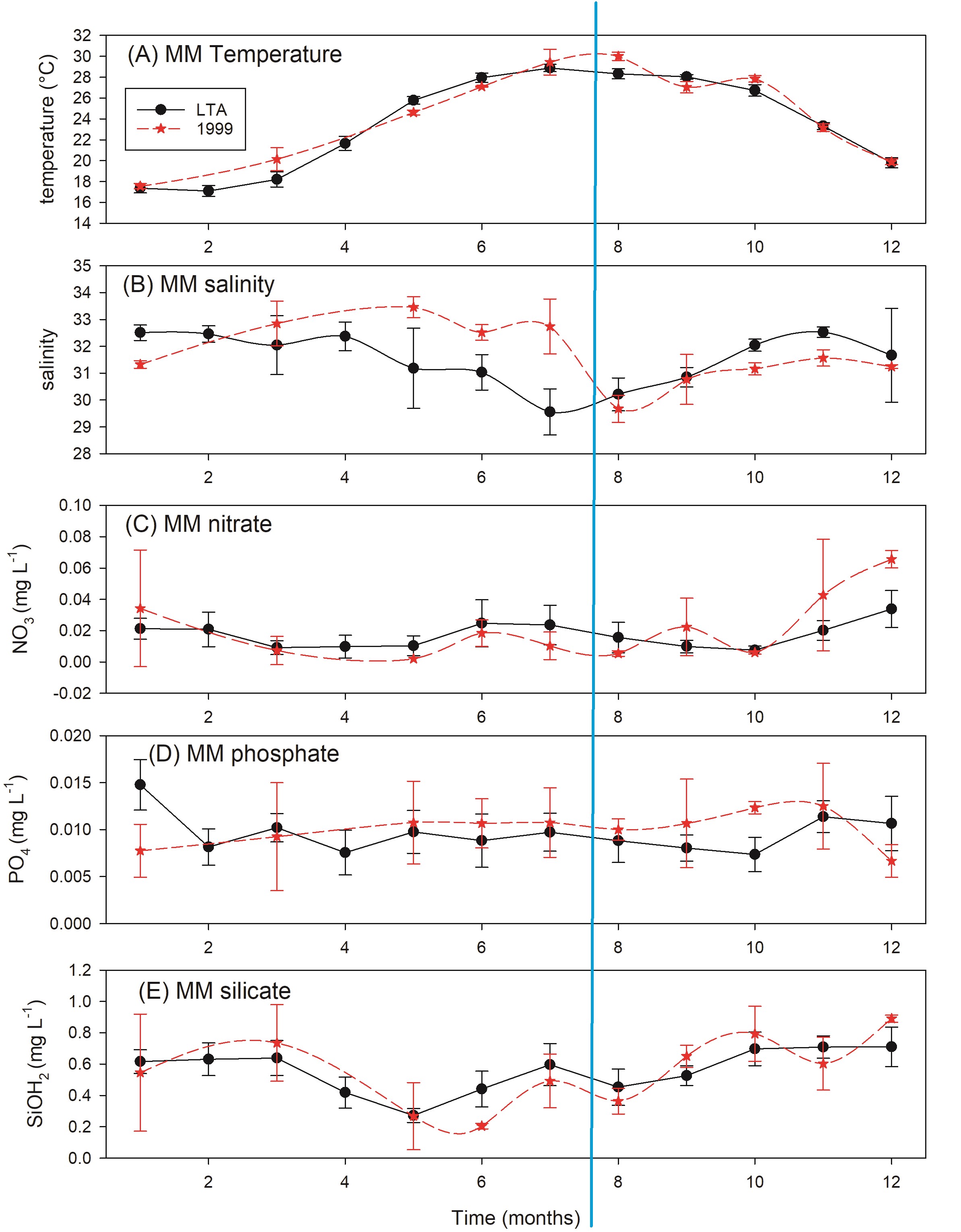

Supplement: FigS17_fbac062 [file figs17_fbac062.jpeg]

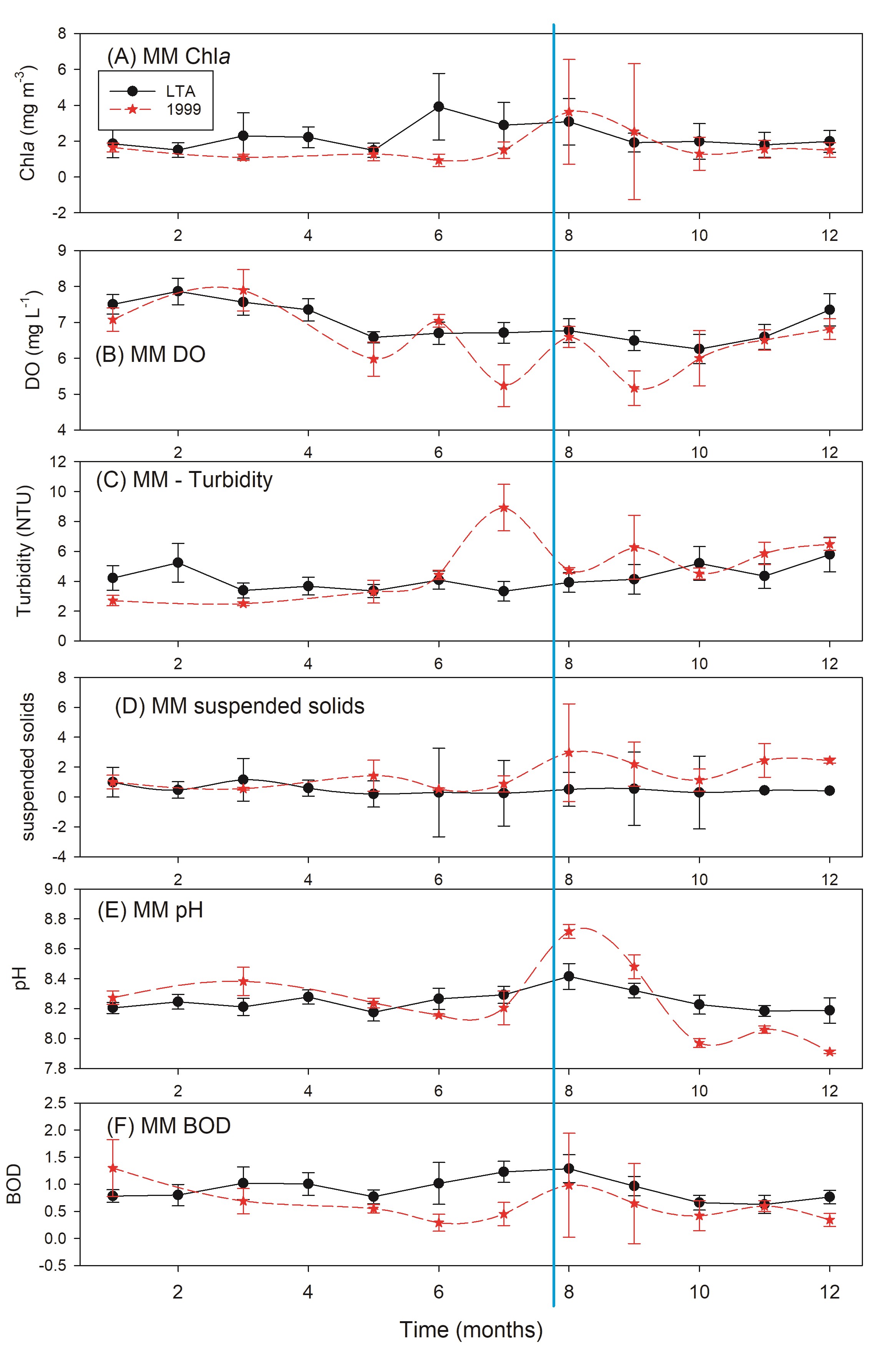

Supplement: FigS18_fbac062 [file figs18_fbac062.jpeg]

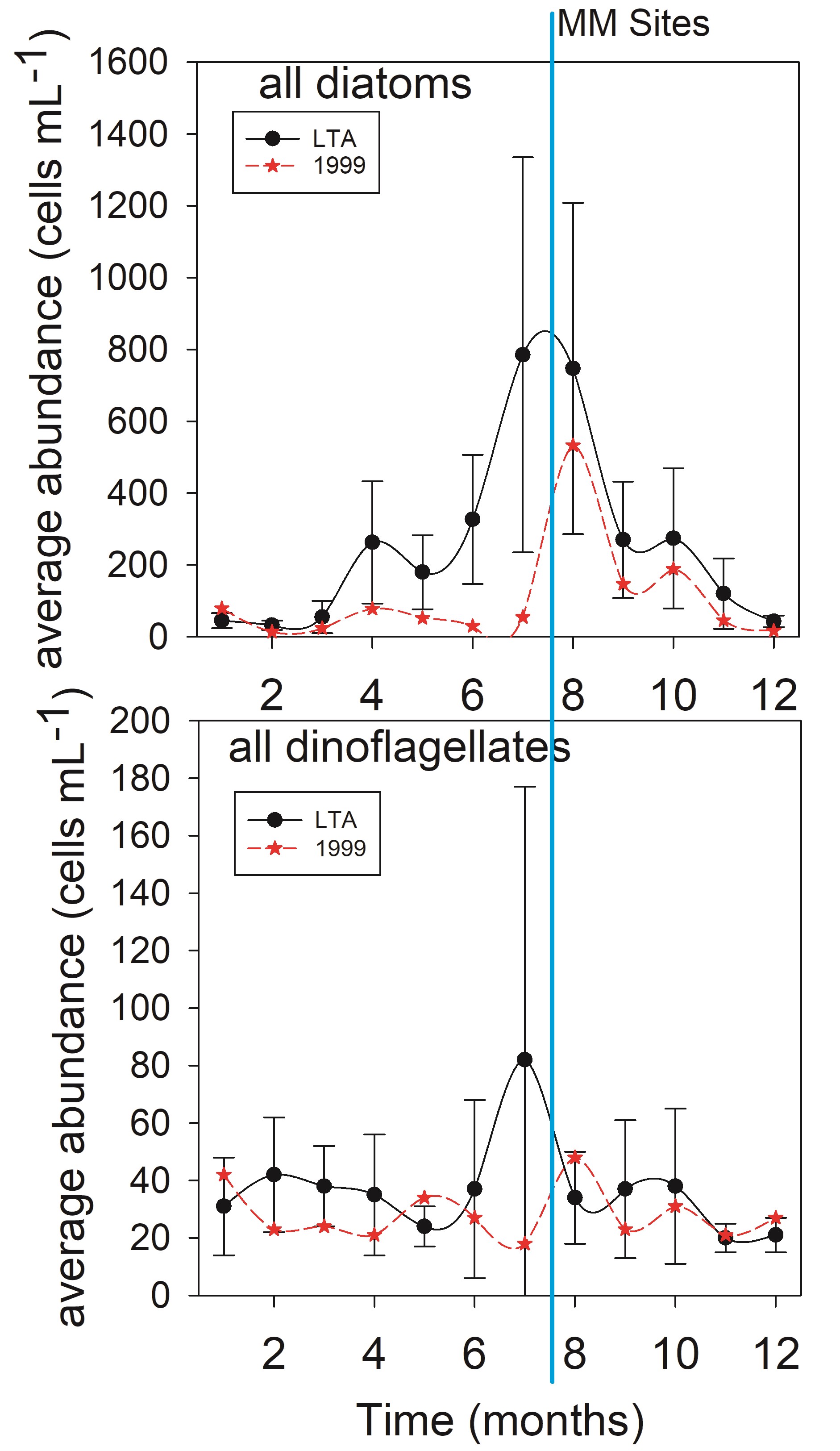

Supplement: FigS19_fbac062 [file figs19_fbac062.jpeg]
